# Do G4 ligands induce mitochondrial dysfunction without ROS induction

**DOI:** 10.1016/j.isci.2026.116036

**Published:** 2026-05-22

**Authors:** Xinru Zhang, Fei Li, Siyi Zeng, Dongyin Lian, Qi Cheng, Lin Li, Xin Gao, Qiantao Wang, Yumei Qin, Junrong Du, Qiang Li, Zhenlei Song, Qingrong Qi, Anton Granzhan, Sophie Bombard, Tao Jia (贾涛)

**Affiliations:** 1 Key Laboratory of Drug-Targeting and Drug Delivery System of the Education Ministry and Sichuan Province, Sichuan Engineering Laboratory for Plant-Sourced Drug and Sichuan Research Center for Drug Precision Industrial Technology, West China School of Pharmacy, Sichuan University, Chengdu 610041, China; 2 NHC Key Laboratory of Nuclear Technology Medical Transformation, Sichuan Clinical Research Center for Radiation and Therapy, Sichuan Provincial Engineering Research Center of Nuclear Medical Equipment Translation and Application, Department of Nuclear Medicine, Mianyang Central Hospital, Mianyang 621000, China; 3 Department of Respiratory Medicine, Mianyang Central Hospital, Mianyang 621000, China; 4 Department of Thoracic Surgery, Sichuan Cancer Hospital, School of Medicine, University of Electronic Science and Technology of China, Chengdu 610041, China; 5 Université Paris Cité, LCBPT, CNRS UMR8601, 75006 Paris, France; 6 CNRS-UMR9187, INSERM U1196, Institut Curie, PSL-Research University, 91405 Orsay, France; 7 CNRS-UMR9187, INSERM U1196, Institut Curie, Université Paris Saclay, 91405 Orsay, France

**Keywords:** Biochemistry, molecular biology, cell biology

## Abstract

G-quadruplex (G4) DNA structures, present in both nuclear and mitochondrial genomes, represent emerging therapeutic targets. Here, we developed and exploited a mitochondrial high-content profiling platform (Mito-HiCP), integrating automated microscopy with quantitative image analysis, to compare the effects of G4 ligands with cisplatin in cancer and normal cells. G4 ligands induce mitochondrial depolarization without reactive oxygen species (ROS) bursts, in contrast to the ROS-associated toxicity of cisplatin. Notably, the G4 ligand 360 A suppresses both mitochondrial and cytosolic ROS levels while retaining tumor selectivity, whereas its non-G4-binding isomer (EDL21) shows no effect, supporting a G4-dependent mechanism. Mechanistically, 360 A reduces mitochondrial DNA copy number, transcription, and electron transport chain protein levels. Using Mito-HiCP, we identify that G4 targeting uncouples mitochondrial membrane potential from ROS production. This reveals a mode of redox regulation that underlies selective anticancer activity and provides a framework to dissect mitochondrial responses and redox regulation across diverse therapeutic perturbations.

## Highlights

• Mito-HiCP quantitatively profiles mitochondrial function and redox states
• G4 ligands depolarize mitochondria without ROS bursts
• MMP-mtROS uncoupling reveals tumor-selective vulnerability
• 360 A drives ROS suppression and mitochondrial dysfunction

## Graphical abstract

**Figure:**
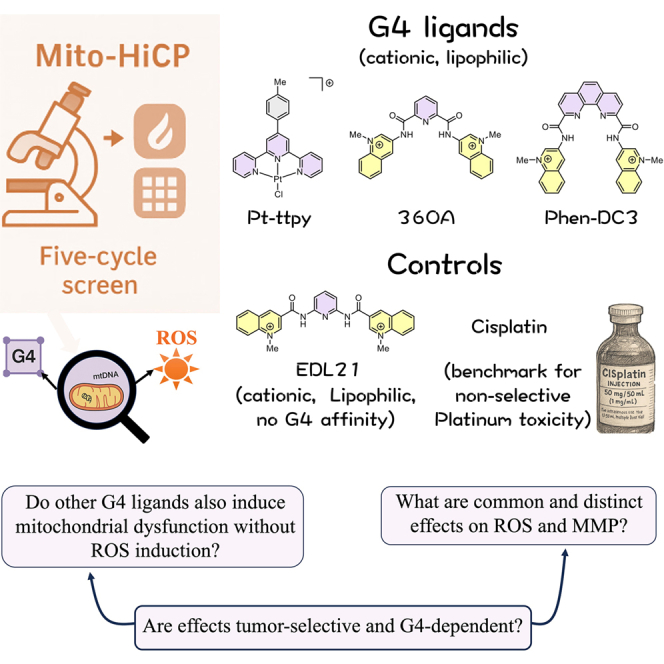

## Introduction

Cancer remains a major global health challenge despite advances in treatment. A promising new strategy is the therapeutic targeting of G-quadruplex (G4) structures, unique four-stranded nucleic acid conformations in guanine-rich DNA or RNA.^1^ G4 structures are not only prevalent in nuclear genomes but also present in mitochondrial DNA (mtDNA).^2^ These structures can influence a range of oncogenic functions through dynamic conformational changes, making them intriguing targets for cancer therapy.^3^ Despite their potential, the effects of G4-targeting therapies on mitochondrial function, reactive oxygen species (ROS) production, and underlying mechanisms, remain largely unexplored.^4^ Even studies have identified 111 potential G4-forming sequences within mtDNA,^5^ there remains a scarcity of small-molecule ligands that specifically target G4-mtDNA to modulate mitochondrial metabolism and related cell death.^6^

To date, several G4 ligands have been reported to accumulate in mitochondria, yet only a subset has been mechanistically demonstrated to interfere with G4-mtDNA. Classical DNA binders such as the cationic porphyrin TMPyP4 and the cyanine dye DODC show mitochondrial accumulation and can induce mitochondrial dysfunction,^7^ although direct G4-mtDNA targets remain unidentified. By contrast, RHPS4 preferentially localizes to mitochondria at low doses, stabilizes mtG4s, inhibits mitochondrial transcription elongation, and reduces mtDNA copy number^8^; Moreover, engineered BMVC-12C-P derivatives directly visualize mtG4 in live cells and suppress mitochondrial gene expression via mtG4 stabilization.^9^ Collectively, previous studies discriminate ligands that merely enter mitochondria (e.g., TMPyP4 and DODC) from those validated to act on G4-mtDNA (e.g., RHPS4 and BMVC-12C-P). However, despite these advances, their targeting specificity in living cells remains limited, and systematic exploration of chemical scaffold for designing bona fide G4-mtDNA-targeting ligands is still scarce.

G-quadruplex (G4) ligands such as 360 A and Phen-DC3 are among the most extensively studied compounds due to their strong and selective stabilization of multiple G4 structures *in vitro.*^10^^,^^11^ Both share the bisquinolinium scaffold, and their anticancer activity has been largely attributed to G4 targeting. For instance, 360 A destabilizes telomere homeostasis,^12^ promotes degradation of the 3′ telomeric overhang, and triggers ATM-dependent DNA damage signaling, leading to repression of oncogenes such as *c-MYC*^13^ and selective cancer cell killing.^14^ By contrast, inversion of the amide connectivity within this scaffold abolishes G4 binding affinity, as demonstrated by the compound EDL21,^15^ which exhibits a Δ*T*_½_ value of <5°C in FRET melting assays, indicative of only minimal G-quadruplex stabilization. Accordingly, we selected EDL21 (Scheme 1) as a negative control in our experiments.

Despite these advances, most studies have concentrated on the nuclear actions of G4 ligands, leaving their mitochondrial roles largely unexplored. This represents a critical gap, as mitochondrial DNA (mtDNA) encodes essential subunits of the respiratory chain, directly linking to mitochondrial membrane potential (MMP) and ROS generation. Binding of G4 ligands to G4-forming sequences in mtDNA could impact transcription and replication,^8^^,^^16^ thereby probably influencing both MMP and ROS. However, few studies have addressed how G4 ligands affect MMP, total ROS, or mitochondrial ROS (mtROS). A recent study introduced a novel dicationic lipophilic ligand that targeted both MMP and mtDNA G4, showing potent anticancer activity through ROS induction and mitochondrial death signaling.^6^ However, whether these effects stemmed from mtDNA targeting, MMP disruption, or a synergistic combination remained unresolved.

We recently demonstrated that Pt-ttpy, a G4-binding platinum(II) complex, selectively targets G4-enriched regions in both nuclear and mitochondrial genomes without generating ROS or mtROS, induces profound mitochondrial toxicity, while its low-affinity analog Pt-tpy is largely inactive.^16^^,^^17^^,^^18^ Our recent findings prompted to investigate whether other canonical G4-stabilizing ligands also induce mitochondrial dysfunction without ROS induction, to delineate their shared and distinct effects, and to determine how these effects differ between cancer and normal cells.

Currently, the G4-ligands database (https://www.g4ldb.org) includes entries for roughly 1,000 small molecules.^19^ The choice of ligands was based on their distinct structural scaffolds, representative binding modes to G4 DNA, and reported biological activities. EDL21 was included as a newly synthesized negative-control compound (poly cationic and lipophilic) structurally related to potential “mitochondrial binder” but lack G4 affinity,^15^ thereby serving as an ideal internal benchmark for G4-mtDNA-dependent effects. Pt-ttpy, a platinum (II) complex, has been reported to dual-target both nuclear and mitochondrial G4 structures, selectively impairing tumor cell mitochondrial function without ROS induction. 360 A and Phen-DC3 are well-characterized, high-affinity G4 ligands with distinct chemical frameworks (bisquinolinium derivatives of pyridine and phenanthroline, respectively), representing prototypical small-molecule G4 stabilizers.^20^ Cisplatin was also chosen as a clinically relevant comparator that induces DNA crosslinks without G4 selectivity, thus enabling discrimination between general DNA damage responses and G4-specific effects; it also provided a well-defined positive control for (mt)ROS induction, serving to validate the responsiveness of the Mito-HiCP system. To further confirm assay robustness, complementary flow cytometry (FACS) analyses were conducted in similar settings. Notably, although RHPS4 is one of the most extensively studied ligands with documented mitochondrial accumulation and direct mtDNA G4 engagement,^2^^,^^5^ it was deliberately excluded from the Mito-HiCP assay because of its strong intrinsic fluorescence overlapping with the spectral range of the live-cell probes used, which resulted in severe signal interference and precluded reliable multiparametric quantification (data not shown).

**Figure 1:**
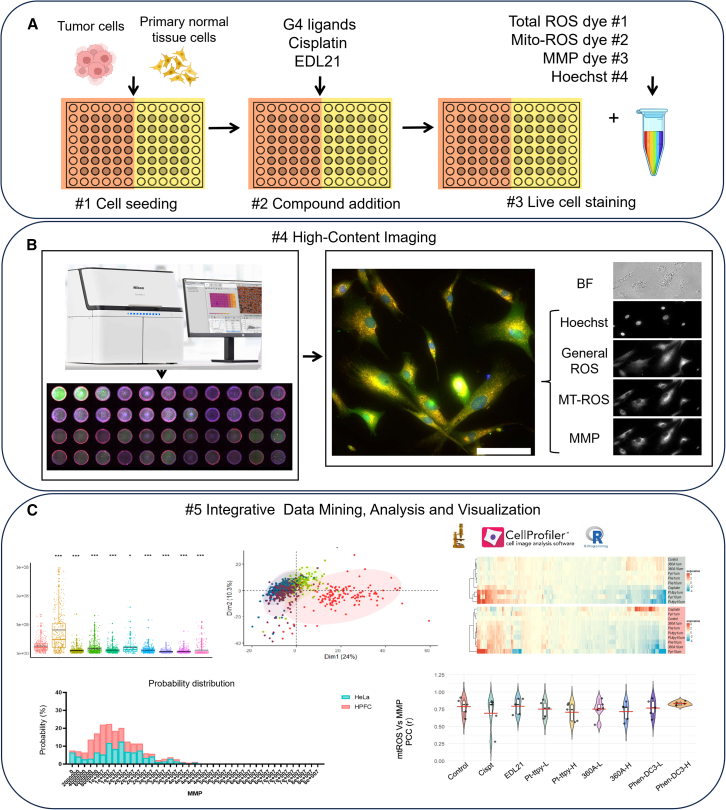
Pipeline of high-content microscopy-based method coupled with combined single-cell Fiji, CellProfiler analysis (Mito-HiCP) to evaluate the impact of different G4 ligands and cisplatin on mitochondria (A) Step #1 illustrates the preparatory procedures before high-content imaging, including the seeding of tumor cells and normal HPFCs in specialized 96-well plates. Step #2 involves the addition of the indicated compounds, including G4 ligands and cisplatin. Step 3 covers the preparation of dye mixtures for live cell staining. (B) Left: Automated high-content imaging is performed using 96-well plates. Right: A representative image of showing four fluorescence channels: Hoechst for nuclear staining, general ROS, mitochondrial ROS (mtROS), and MMP for mitochondrial health. Bright field (BF) imaging is also collected during the screening, both for precise automated localization of cells during image collection and as a quick reference for assessing cell health status. (C) The analysis workflow integrates free software platforms including Fiji, CellProfiler, and R for imaging data mining, analysis, and visualization. See also Figures S14 and S15.

To address this, we selected three well-characterized, high-affinity G4 ligands (Pt-ttpy, 360 A, and Phen-DC3), together with cisplatin and the negative control EDL21, a cationic, lipophilic compound lacking G4 affinity. We then established an integrated high-content microscopy pipeline that combines automated Fiji-based single-cell fluorescence quantification with CellProfiler-based morphological profiling to systematically evaluate their effects in human primary fibroblast cells (HPFCs) and cancer cells HeLa. We termed this platform Mito-HiCP (mitochondrial high-content cell profiling) (Figure 1). By integrating high-content imaging, quantitative fluorescence analysis, and multiparametric morphological profiling, Mito-HiCP enables the simultaneous assessment of mitochondrial structure and function, and cellular redox status using a panel of dyes for total ROS, mitochondrial ROS, and MMP.

Using Mito-HiCP, we sought to clarify how G4 ligands and functional control compounds influence mitochondrial health. Fiji analysis macros (Data S1), RStudio visualization scripts (Data S4 and S5), and detailed CellProfiler segmentation and downstream analysis workflows (Data S2 and S3 and Document S1) are provided in the supplementary materials. This approach enabled us to delineate both the shared and distinct effects of different G4 ligands, uncover mechanisms linking their actions to ROS, mtROS, and MMP regulation, and address the unexplored gap in understanding how G4-targeting compounds modulate mitochondrial function in cancer versus normal cells.

## Results

### Single-cell quantification of the effect of G4 ligands and control compounds on ROS, mtROS, and MMP in HeLa and HPFC cells

We assessed the mitochondrial effects of G4 ligands, with cisplatin serving as a positive control for ROS induction and mitochondrial toxicity. Bright field (BF) imaging (Figure S1) was performed to monitor overall cell status, while DCFH-DA, MitoSOX Deep Red, and TMRE were applied to measure total ROS, mitochondrial ROS (mtROS) and morphology, and MMP, respectively (Figure 1; Table S1). To control for nonspecific physicochemical effects, we employed EDL21, a lipophilic compound structurally similar to 360 A but lacking G4 affinity, as a negative control (Scheme S1).

**Figure 2:**
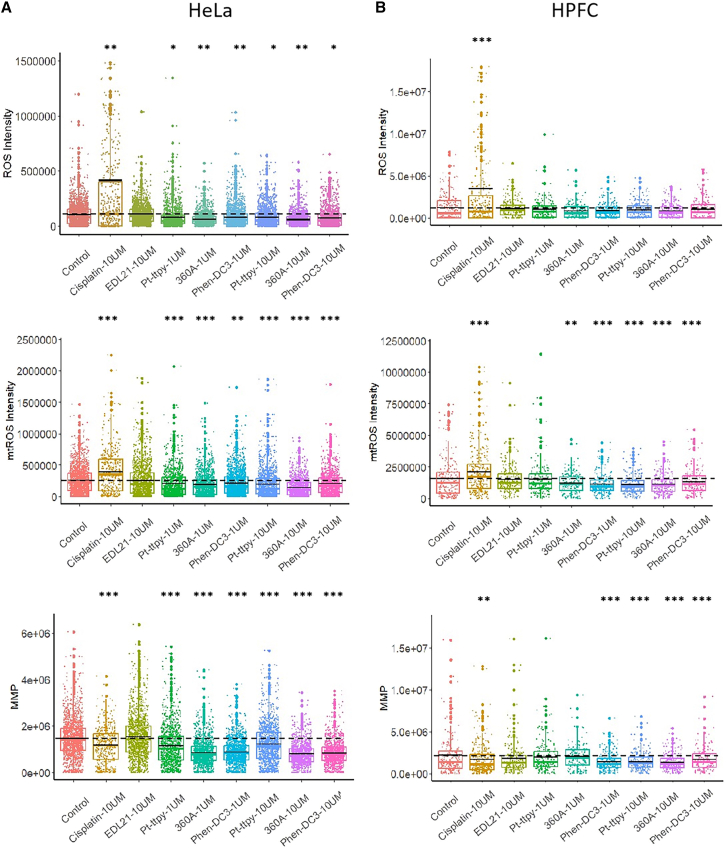
Single-cell fluorescence quantification of the effects of G4 ligands, cisplatin, and EDL21 on mitochondrial membrane potential (MMP), total ROS, and mitochondrial ROS (mtROS) in cancer cell line HeLa (A) and human primary normal fibroblast cells HPFC. (B) cells. Graphs show quantification of ROS, mtROS, and MMP levels after 24 h of treatment with the indicated compounds. Statistical significance was assessed using an unpaired Student’s *t* test, comparing each treatment group to the control. ∗*p* < 0.05; ∗∗*p* < 0.01; ∗∗∗*p* < 0.001. Each group represents data from at least 200 cells. Colored horizontal lines indicate the median, black lines indicate the mean, and dashed lines denote the mean of the control group. Data represent five independent experiments acquired through high-content microscopy screening. See also Figures 1, 2, 3, 4, and 5.

**Figure 3:**
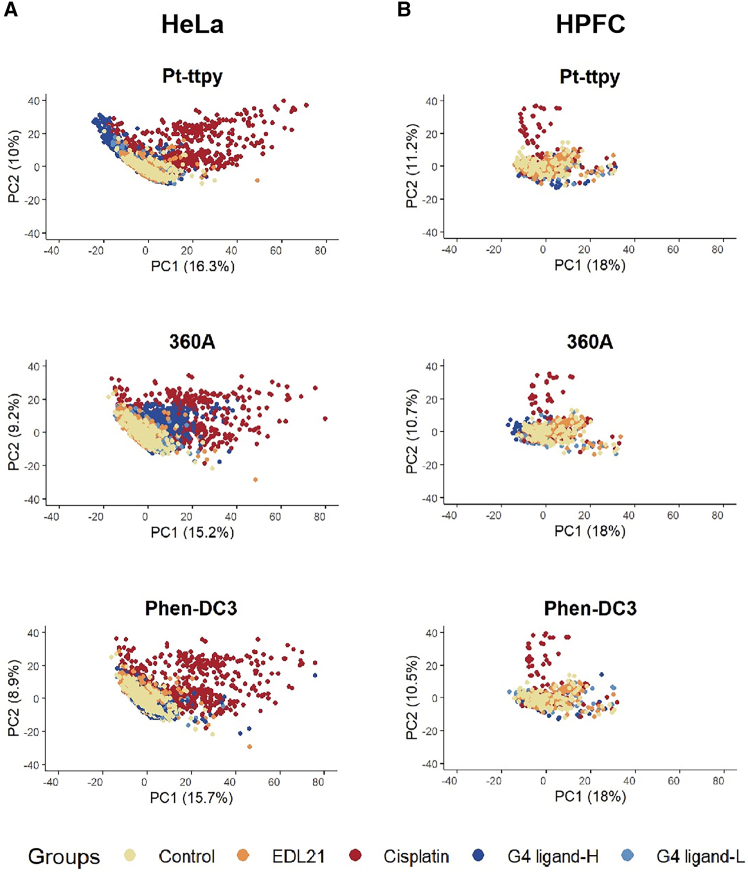
Dimensionality reduction and phenotypic clustering differentiate cells’ responses to different G4 Ligands and functional control compounds, as well as control group in both cancer (A) and normal cells (B). Principal-component analysis (PCA) was performed on CellProfiler-derived features from cellular images of HeLa and HPFC cells treated for 24 h with G4 ligands (360 A, Phen-DC3, Pt-ttpy at low 1 μM and high 10 μM concentrations), cisplatin (10 μM), or EDL21 (10 μM). (A and B) PCA plots depict sample distributions across treatment groups, categorized as control, EDL21, cisplatin, G4 ligand-L (Low con.), and G4 ligand-H (High con.). Each dot represents the mean feature values of all cellular objects within a single image. The percentage of variance explained is shown on each axis. See also Figures S8–S10.

In HeLa cancer cells, cisplatin (10 μM) markedly increased both ROS and mtROS levels, accompanied by a pronounced loss of MMP. By contrast, all tested G4 ligands induced a significant reduction of MMP without elevating ROS, (Figure 2A). Notably, a slight but significant suppression of ROS and mtROS production has been found and confirmed by flow cytometry (Figures 2 and 3). At the same time, EDL21 had no detectable effect on MMP, ROS, or mtROS, excluding nonspecific influences of lipophilicity or uptake and suggesting a G4-specific mechanism. These results demonstrate that these G4 ligands impair mitochondrial function in cancer cells while avoiding ROS overproduction that is dependent on G4-binding properties.

In human primary fibroblast cells (HPFC), cisplatin also triggered a sharp rise in ROS and mtROS with severe MMP collapse, indicating non-selective mitochondrial toxicity. By contrast, Pt-ttpy and 360 A at low doses (1 μM) caused minimal MMP collapse in HPFCs (Figure 2B), highlighting their cancer-selective activity. Consistently, G4 ligands showed negligible effects on ROS in HPFCs. Extended viability assays further revealed differential cytotoxicity: 360 A (10 μM, 72 h) selectively killed HeLa cells, with similar effects observed in another cancer cells H226 from lower dose of 5 μM to high doses of 50 μM (Figure S4). In contrast to all G4 ligands and cisplatin, EDL21 show no toxicities to cancer cells (Figure S5).

Our previous work indicates Pt-ttpy targets mitochondrial G4 structures,^16^^,^^17^ while herein EDL21, lacking G4 affinity, failed to alter ROS, mtROS, or MMP, thereby suggesting the requirement of G4 binding for impairing mitochondrial function. Additional experiments (Figure S6) showed that cancer cell line HeLa display significantly higher basal MMP and mtDNA transcriptional activity than normal primary cells HPFC. Cancer cells’ elevated metabolic demand necessitates more frequent mtDNA transcription and replication, process that naturally give rise to transient G4 structures. Cancer cells therefore become increasingly dependent on the regulated resolution of mtG4s to maintain MT genomic stability, rendering them more vulnerable when that structures are pharmacologically stabilized. In addition, the intrinsic mitochondrial vulnerability, coupled with the characteristically higher mtDNA mutation burden (e.g., POLG defects) in cancer cells, which compromises replication fidelity and repair capacity.^21^ Together, these factors likely act synergistically to account for the selective sensitivity of cancer cells to G4-targeting interventions, in the perspective of mitochondrial G4-DNA targeting strategies.

Collectively, these findings demonstrate that all tested G4 ligands exhibited a tendency to selectively depolarize mitochondria and exert cytotoxic effects in cancer cells, with 360 A showing the most pronounced activity. In HeLa cells, not HPFC normal cells, all G4 ligands caused significant modulation of general ROS (Figure 2A), while Pt-ttpy and 360 A at low concentrations preferentially reduced MMP in tumor cells but not in normal cells, with 360 A the most potent even at 1 μM (Figures 2A and 2B bottom; Figure S4), indicating selective mitochondrial toxicity. In contrast, cisplatin produced a non-selective mitochondrial toxicity, as evidenced by a marked decrease in MMP accompanied by robust ROS induction in both cells, thereby lacking tumor selectivity. EDL21 showed no detectable effects across all readouts, validating its role as a negative control. Notably, all the tested cationic and lipophilic G4 ligands reduced, rather than induced, ROS levels, which distinguishes their mechanism from that of cisplatin. The preferential mitochondrial depolarization observed in HeLa cells compared with HPFCs underscores G4 structures as a cancer-specific vulnerability and highlights the therapeutic potential of G4-targeting strategies.

### Morphological profiling and mechanistic Insights of G4 ligands and control compounds in cancer vs. normal cells

To further dissect the effects of G4 ligands and elucidate the mechanisms underlying their differential mitochondrial toxicity in cancer versus normal cells, we extended our analysis beyond ROS, mtROS, and MMP signal cell intensities to include comprehensive morphological profiling of whole-cell, mitochondrial, and nuclear states. Using a CellProfiler-based pipeline comprising 541 cellular features (Supplementary part in Document S1: feature analysis 1.2.4 and 1.2.(5),^22^ we analyzed spatial relationships between organelles, cross-channel correlations, and staining texture metrics. In principal-component analysis (PCA) analysis, we found the features contributing most significantly were identified as texture and intensity (Figure S7). PCA and hierarchical clustering heatmaps (Figure 3; Figure S8) revealed both shared and distinct phenotypic signatures induced by different G4 ligands compared with cisplatin and EDL21. These analyses enabled discrimination of G4-dependent phenotypes from nonspecific physicochemical or ROS-induction-related cytotoxic effects, providing a systems-level perspective on how G4 stabilization uniquely drives coordinated alterations in mitochondrial morphology, nuclear organization, and cellular redox state.

Cancer vs. normal cell profiles are as follows: In HeLa cells, G4 ligands produced phenotypic distributions clearly separated from controls and EDL21, and distinct from cisplatin (Figures 3A, S8, and S9). At low concentrations (1 μM), G4 ligands displayed highly similar effects, whereas higher doses (10 μM) led to divergent patterns but still markedly different from cisplatin, indicating a unique, non-ROS-dependent mechanism. By contrast, in HPFCs, G4 ligands at both concentrations overlapped extensively with controls and EDL21 (Figures 3B, S8, and S9), highlighting their minimal impact in normal cells. These differences may arise from intrinsic features, particularly the higher baseline MMP in HeLa compared with HPFCs, despite comparable mtDNA copy numbers (Figure S6B).

Similarities and differences between the G4 Ligands are as follows: heatmap clustering (Figure S10) further highlighted both commonalities and distinctions. In HeLa cells, cisplatin and Pt-ttpy formed separate clusters associated with strong mitochondrial toxicity, whereas 360 A and Phen-DC3 clustered closely, consistent with their shared bisquinolinium scaffold. Transcriptomic studies support this observation, as both ligands regulate genes enriched for promoter-proximal G4 motifs.^23^^,^^24^ Interestingly, in normal HPFCs, 360 A and Phen-DC3 also clustered together, but with profiles nearly identical to controls. Collectively, these results confirm that these G4 ligands act through shared non-ROS induction-dependent mechanisms in cancer cells, while still displaying ligand-specific differences in mitochondrial toxicity and (mt)ROS reduction-dependent effects.

G4 stabilization drives tumor-selective mitochondrial and nuclear remodeling beyond general DNA damage, as evidenced by PCA of high-content morphological features. PCA of high-content morphological features revealed distinct cellular phenotypes induced by G4 ligands in tumor (HeLa) versus normal (HPFC) cells (Figure 3). In HeLa cells, high-dose treatments with Pt-ttpy, 360 A, and Phen-DC3 produced extensive shifts along both PC1 and PC2 axes, indicating broad remodeling of mitochondrial and nuclear morphology. Among them, 360 A elicited the most pronounced phenotypic divergence, forming a cluster clearly separated from controls and from the non-G4-binding analog EDL21 (Figure 3A).

In contrast, HPFC cells exhibited tighter clustering with minimal displacement (Figure 3B), suggesting attenuated responses and a higher tolerance to G4-ligands-induced stress. Importantly, cisplatin, despite inducing cytotoxic stress in both cell types (Figures S1–S5), caused only moderate phenotypic dispersion without generating a distinct cluster comparable to that of G4 ligands in HeLa cells (Figure 3A). This indicates that the morphological heterogeneity and network remodeling observed in tumor cells are not merely due to general DNA damage but rather reflect a G4-specific mitochondrial and nuclear perturbation signature. Together, these data highlight that G4 stabilization triggers stronger and more selective phenotypic remodeling in cancer cells than cisplatin or non-G4 analogs, consistent with the heightened mitochondrial vulnerability of tumor cells to G4-targeting interventions.

Collectively, morphological profiling findings demonstrate that G4 ligands induce distinctive and coordinated alterations in mitochondrial and nuclear/cellular morphology that are largely confined to cancer cells. Morphological profiling clearly separated G4-ligand-treated HeLa cells from controls and from both cisplatin and the non-G4-binding analog EDL21, while normal HPFC cells remained largely unaffected even at higher ligand concentrations. Among the tested compounds, G4 ligand 360 A consistently produced the most pronounced phenotypic divergence, correlating with its strong mitochondrial depolarization and phenotypical studies as shown in Result 1 and Figures S1, S2–S5, and S8. This reinforces that 360 A exerts a higher tumor-selective toxicity, reflecting its ability to selectively destabilize mitochondrial homeostasis in malignant cells without eliciting comparable perturbations in normal counterparts. Taken together, the integrated morphological, functional, and mechanistic analyses support a model in which G4 stabilization, particularly by 360 A, drives cancer-specific cell remodeling, revealing a distinct vulnerability axis as compared with cisplatin.

**Figure 4:**
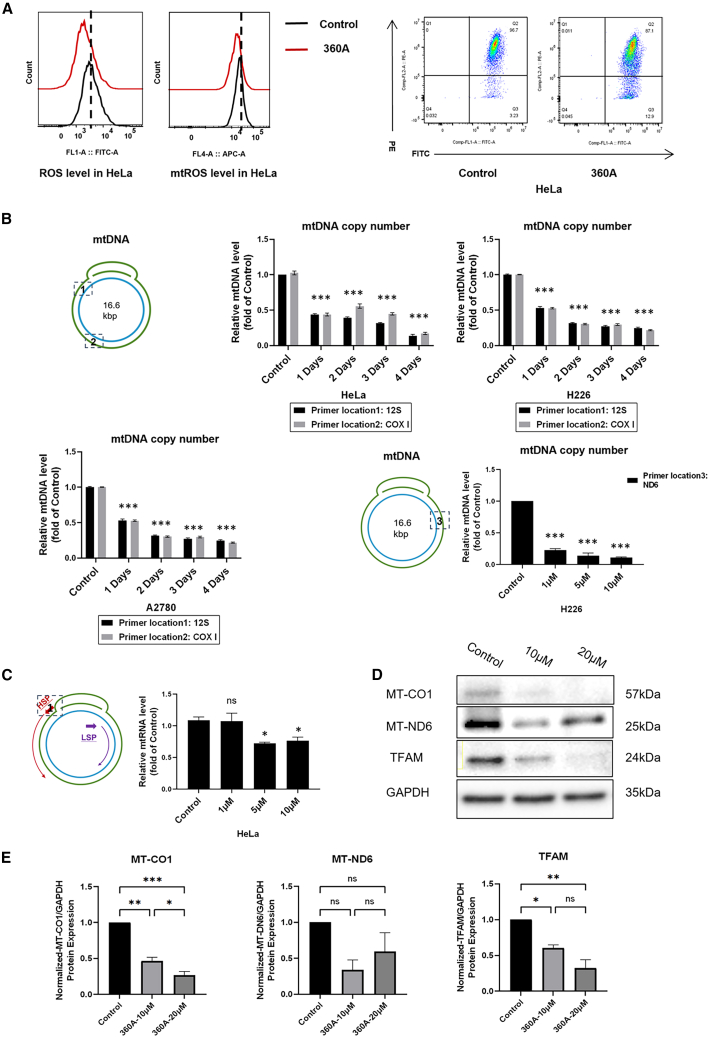
360 A reduces mtDNA abundance and mitochondrial gene expression in cancer cells without ROS induction (A) Flow cytometry analysis showing that treatment with 360 A reduced mitochondrial ROS (mtROS) and total cellular ROS in HeLa cells (10 μM, 1 day) and decreased mitochondrial membrane potential as indicated by reduced JC-1 fluorescence. Quantification is shown in Figures S2 and S3. Data represent at least two independent experiments. (B) Schematic illustrating primer locations used for qPCR quantification of mtDNA copy numbers. Time-course analysis of mtDNA content in HeLa, H226, and A2780 cells treated with 360 A (15 μM, 1–4 days), measured by qPCR targeting mitochondrial 12 S rRNA and COX I and normalized to the nuclear gene β-actin. Dose-response analysis of mtDNA abundance (MT-ND6) in H226 cells treated with 360 A (1–10 μM, 3 days). Data are shown as mean ± SEM (*n* = 2). Statistical significance was determined by one-way ANOVA versus control (∗∗∗*p* < 0.001). (C) Relative mitochondrial transcript levels (12 S rRNA) in HeLa cells treated with 360 A (1–10 μM, 3 days), normalized to β-actin. Data are shown as mean ± SEM (*n* = 2). Statistical significance was determined by unpaired *t* test versus control (∗*p* < 0.05; ns, not significant). (D) Western blot analysis of TFAM and mitochondrially encoded proteins (MT-ND6 and MT-CO1) in HeLa cells treated with 360 A (10 and 20 μM, 3 days). GAPDH was used as a loading control. (E) Quantification of protein levels in (D), normalized to GAPDH and expressed relative to control. Data are shown as mean ± SEM (*n* = 2). Statistical significance was determined by one-way ANOVA (∗*p* < 0.05, ∗∗*p* < 0.01, ∗∗∗*p* < 0.001; ns, not significant).

360 A suppresses (mt)ROS and MMP, induces mtDNA depletion and consequent protein repression. Next, we further investigated the mechanism underlying 360 A-induced mitochondrial dysfunction in cancer cells without ROS induction. As shown in Figure 4A did not trigger ROS bursts, as indicated by the ROS (DCFH-DA) assay, but instead significantly reduced both mitochondrial and cytosolic ROS levels while inducing marked MMP perturbation. Quantitative analysis of all tested compounds is provided in Figures 2, 3, and 4 and is consistent with the Mito-HiCP screening results (Figure 2).

mtDNA copy number was quantified by qPCR following protocol described in our previous work.^16^ Distinct primer sets were used, and primer locations are shown in Figure 4B. Thus, we next quantified mitochondrial DNA (mtDNA) copy number, across multiple cancer cell lines following 360 A treatment, and observed a consistent and significant reduction in mtDNA abundance (Figure 4B), providing direct evidence that the G4 ligand exposure compromises mitochondrial genome maintenance. In HeLa cells, mitochondrial RNA analysis revealed marked inhibition of mitochondrial gene transcription (Figure 4C), accompanied by a substantial reduction in key electron transport chain (ETC) proteins, including COX1 and ND6 (Figure 4D). These results directly link G4 ligand treatment to impaired mitochondrial gene expression and respiratory chain integrity.

Notably, 360 A also caused a pronounced decrease in the level of TFAM protein, a nuclear-encoded factor essential for mtDNA replication and transcription that is imported into mitochondria. This observation is consistent with our recent findings for Pt-ttpy and suggests that G4 ligands, including 360 A and Pt-ttpy, perturb mitochondrial function through combined mitochondrial and nuclear G4-dependent mechanisms.^16^

Taken together, these data demonstrate that G4 ligand 360 A induces mtDNA depletion and transcriptional impairment, leading to reduced levels of ETC proteins and mitochondrial depolarization without ROS induction, but rather with an overall reduction in (mt)ROS levels. This ROS-independent mode of mitochondrial dysfunction provides a mechanistic basis for the tumor-selective mitochondrial vulnerability revealed by Mito-HiCP profiling.

**Figure 5:**
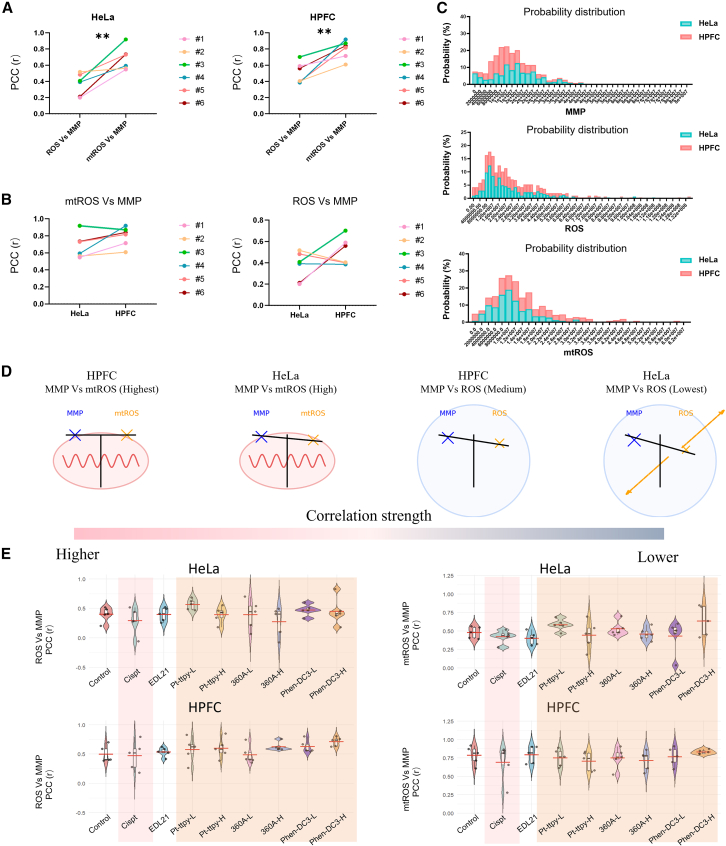
Comparative correlation analysis and distribution of ROS, mtROS, and MMP in normal and tumor cell models Correlations were assessed using Pearson’s correlation coefficient (r) as the vertical axis, represented as PCC (r). (A) Correlation plots of ROS versus MMP and mtROS versus MMP in HeLa (left) and HPFC (right) cells. Statistical significance was assessed using an unpaired Student’s *t* test, comparing each treatment group to the control. ∗∗*p* < 0.01. (B) Comparative analyses of mtROS-MMP (left) and ROS-MMP (right) correlations in HeLa and HPFC cells. (C) Distribution of single-cell intensities for MMP, ROS, and mtROS in HeLa and HPFC cells. (D) Schematic illustration of correlation changes between ROS and MMP, as well as mtROS and MMP, in HeLa versus HPFC cells (Table S3). (E) Correlation distributions of ROS-MMP and mtROS-MMP in HeLa and HPFC cells treated with cisplatin, EDL21, or different G4 ligands. Violin plots show the distribution of PCC (r) values. Black dots represent individual data points. Embedded boxplots indicate the interquartile range, with the center line representing the median. Red horizontal lines denote the mean value of each treatment group. See also Figures S11–S13.

Correlation of MMP and (mt) ROS in cancer vs. normal cells are as follows: We next examined whether the selective action of G4 ligands is associated with differences in mitochondrial homeostasis, as reflected by alterations in the correlation between MMP and either total ROS or mitochondrial ROS (mtROS). At baseline, both HeLa and HPFC cells displayed a markedly stronger correlation between MMP and mtROS than between MMP and total ROS (Figure 5A). Across six independent experiments, the MMP-ROS correlations were consistently slightly higher in HPFC cells than in HeLa cells (Figure 5B; Figure S11; Table S3). To compare correlation strengths, we further performed Fisher’s *z*-tests. In both cell lines, the correlation between MMP and total ROS was significantly weaker than that between MMP and mtROS (HeLa: *z* = −2.131, *p* = 0.017; HPFC: *z* = −11.1, *p* ≤ 0.001). Moreover, the mtROS-MMP correlation was significantly stronger in HPFC cells than in HeLa cells (*z* = 3.827, *p* ≤ 0.001).

According to the “ROS balance” hypothesis,^25^ extreme fluctuations in MMP disrupt electron flow in the ETC or antioxidant enzyme activity, leading to elevated ROS. In normal cells, efficient ETC antioxidant coupling maintains robust mtROS-MMP correlations, whereas in HeLa cells, metabolic rewiring and the Warburg effect decouple ETC function. Although MMP is elevated, increased electron leakage reduces the proportional relationship between mtROS and MMP.^26^^,^^27^ Frequency distributions further confirmed narrower MMP ranges in normal primary cells HPFC, in contrast to broader, flattened distributions in cancer cells HeLa (Figure 5C).

The previous correlation mapping among mitochondrial and cellular oxidative parameters suggested a progressive decoupling of mitochondrial potential and redox homeostasis in cancer cells (Figure 5D). In normal HPFCs, the correlation between MMP and mtROS was the strongest, reflecting a tightly regulated oxidative phosphorylation system and efficient mitochondrial-antioxidant coupling. By contrast, HeLa cells exhibited a marked reduction in MMP-mtROS correlation strength and an even weaker association between MMP and total ROS, indicating disrupted feedback between electron transport and cytosolic ROS buffering. This shift suggests that tumor mitochondria operate at a higher basal potential but with impaired redox control (Figures 5A, 5B, 5C, and S6), probably rendering them more vulnerable to G4-ligands-induced perturbations of mitochondrial homeostasis. Together, these data support the hypothesis that the selective cytotoxicity of G4 ligands, particularly 360 A, arises from their ability to exploit the pre-existing mitochondrial-redox uncoupling in cancer cells, thereby amplifying redox imbalance beyond the adaptive capacity of malignant mitochondria.

Differential effects of G4 ligands, cisplatin, and EDL21 on mitochondrial-redox coupling: To substantiate this hypothesis, we next evaluated how G4 ligands, cisplatin, and the non-G4-binding analog EDL21 influence the correlation strength among mitochondrial potential and (mt)ROS levels, thereby assessing the integrity of mitochondrial-redox coupling in cancer versus normal cells.

Cisplatin treatment markedly reduced MMP-mtROS correlations in both HeLa and HPFC cells, reflecting a broad disruption of mitochondrial homeostasis (Figures 5E, S12, and S13; Table S3). By contrast, G4 ligands induced greater variability in correlation patterns in HeLa cancer cells, while maintaining relatively stable distributions in primary normal cells HPFC. This selective perturbation of mitochondrial coupling in cancer cells could be consistent with their selective toxicity. EDL21, in contrast, caused minimal perturbation, with MMP-mtROS correlation distributions largely overlapping with controls. Interestingly, a modest decrease was detected only in tumor cells, suggesting that its polycationic lipophilic nature may promote mitochondrial accumulation, rendering tumor cells (with higher MMP) more vulnerable than normal cells (Figures S6A and S6B left). However, this nonspecific effect was much weaker than that of G4 ligands and cisplatin. Together, these findings highlight that while polycationic lipophilic accumulation can contribute to mitochondrial perturbation, G4-binding activity provides the critical enhancement that enables G4 ligands to effectively and selectively disrupt mitochondrial homeostasis in cancer cells, at least at low concentration.

### Proposed model

**Figure 6:**
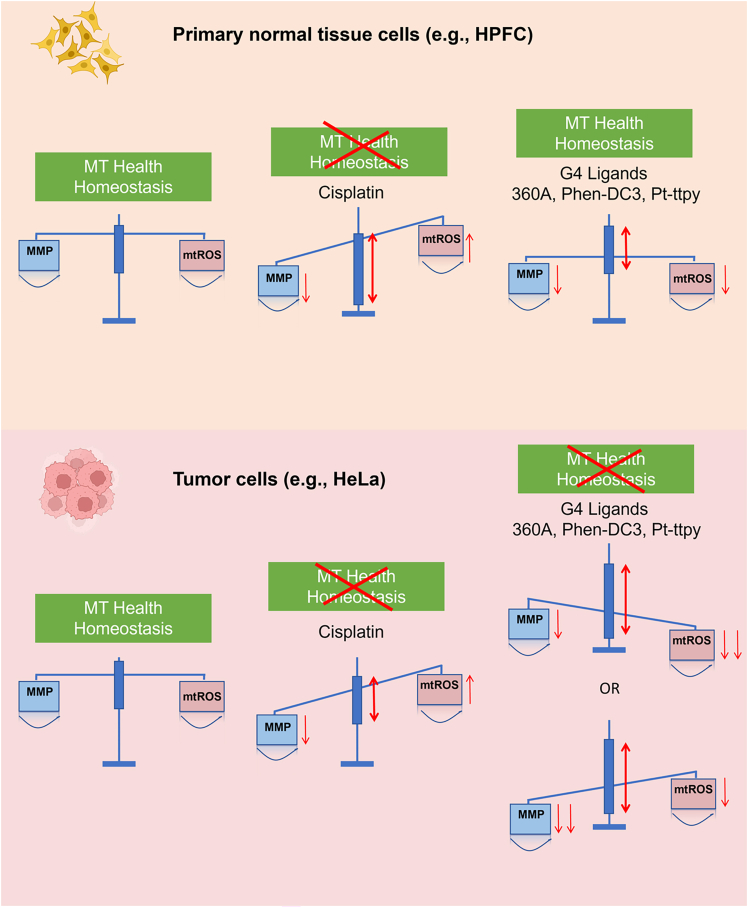
Proposed model of mitochondrial homeostasis and its disruption in primary human primary normal cells and tumor cell, post-treatment of cisplatin and three canonical G4 Ligands (360 A, Phen-DC3, and Pt-ttpy)

Here, we propose a mechanistic model (Figure 6) in which the coupling between MMP and mitochondrial ROS (mtROS) defines a dynamic equilibrium of “mitochondrial health homeostasis.” In this model, cisplatin lowers MMP and provokes widespread oxidative stress, thereby collapsing the mtROS-MMP correlation in both cancer and normal cells and leading to non-selective cytotoxicity. In contrast, G4 ligands, particularly 360 A, selectively modulate this coupling in cancer cells. Rather than inducing ROS bursts, they act in a “regulatory” mode, subtly lowering MMP while maintaining or even reducing ROS levels (Figures 2, 4, 5, and S2–S5). This controlled modulation of the mitochondrial redox network amplifies existing vulnerabilities in tumor mitochondria, thereby triggering cell death without disturbing “redox balance” in normal cells. Such a mechanism underlies the enhanced selectivity and potential safety profile of some G4 ligands compared with classical DNA-damaging agents.

## Discussion

Recent studies have reported newly developed mitochondria-targeted mtG4 ligands that efficiently accumulate in mitochondria, reduce MMP, and concomitantly induce marked ROS production.^6^^,^^28^ This ROS-amplifying phenotype differs from what we observed herein with the canonical, widely used G4 ligands (360 A, Phen-DC3, and Pt-ttpy), which depolarize tumor mitochondria without triggering ROS bursts and, in the case of 360 A, even suppress both cytosolic and mitochondrial ROS. We propose that this divergence primarily reflects the differences in preferential subcellular distribution across G4 ligand classes.

A key distinction is that the canonical ligands tested herein are not designed for exclusive mitochondrial accumulation. Instead, they likely engage a broader nucleic-acid target spectrum across the nuclear genome, mitochondrial genome, and potentially RNA G4s, resulting in a more distributed and comparatively “regulatory” mitochondrial perturbation rather than an acute oxidative injury program. This interpretation is consistent with the prior evidence showing that Pt-ttpy can target G4-enriched regions in both the nucleus and mitochondria,^16^^,^^17^ and with the fact that Phen-DC3 stabilizes RNA G4 structures, supporting a broad cellular target profile.^29^ According to Dumas et al.*,*^29^ RNA G4 regulate outer-mitochondrial-membrane-localize translation of nuclear-encoded mitochondrial proteins, thereby reshaping energy metabolism without inducing oxidative stress. Our findings at the functional single-cell level are consistent with this emerging model, suggesting that the tested canonical G4 ligands primarily act by rewiring mitochondrial protein supply rather than triggering ROS induction-driven damage.

Importantly, the intensity of mitochondrial genome disruption also appears to be G4 ligand-dependent. Falabella et al. showed that Phen-DC3 does not induce mtDNA depletion to the extent observed for RHPS4,^8^ highlighting that not all G4 ligands exert equivalent mtDNA-level effects even when mitochondrial phenotypes overlap. A recent preprint further suggested that 360 A and Phen-DC3 can reduce mtDNA in *P. falciparum*,^30^ but the magnitude of this effect is smaller than that induced by RHPS4; it was proposed that physicochemical properties affecting mitochondrial uptake (e.g., net positive charge and Δψm-driven accumulation) may explain these differences. Together, these data support a framework in which mitochondrial enrichment capacity and target-spectrum breadth jointly shape whether a G4 ligand produces a ROS-amplifying damage phenotype or a ROS-independent regulatory phenotype.

As shown in Figure 4, in multiple tumor cell lines (HeLa, H226, and A2780), 360 A consistently reduced mtDNA copy number and, in HeLa cells, suppressed mitochondrial transcription and reduced ETC components (COX1 and ND6), accompanied by a decreased TFAM protein level. These results provide a mechanistic basis for the ROS-independent mitochondrial dysfunction captured by Mito-HiCP: mtDNA depletion and transcriptional impairment can reduce ETC protein abundance and depolarize mitochondria without requiring ROS amplification. In this context, ROS suppression observed for 360 A may reflect altered respiratory output and/or remodeling of redox buffering, rather than an oxidative burst-driven mitochondrial injury cascade. Collectively, these findings suggest that whether a G4 ligand induces mitochondrial ROS bursts is not determined solely by G4-binding ability but instead depends on (1) mitochondrial accumulation strength, (2) subcellular distribution, and (3) the breadth of G4 targets engaged (mtDNA, nuclear DNA, and possibly RNA). Mitochondria-targeted mtG4 ligands that accumulate strongly can impose intense local stress, favoring electron leakage and ROS elevation. In contrast, canonical non-mitochondria-selective ligands act through a broader cellular distribution, and in our datasets, preferentially manifest a ROS-independent mode of tumor-selective mitochondrial dysfunction. This mechanism should be regarded as a testable hypothesis, rather than a definitive conclusion. A more refined stratification of G4 ligands, beyond simple mitochondrial versus non-mitochondrial targeting, will likely emerge from systematic analyses encompassing a wider chemical space, including the ligands with diverse physicochemical properties and both natural and synthetic G4-binding scaffolds. In this context, Mito-HiCP provides a scalable platform to interrogate how distinct classes of G4 ligands differentially reshape mitochondrial homeostasis and redox control in future studies.

To the best of our knowledge, the Mito-HiCP platform provides the first systematic evidence that canonical G4 ligands selectively impair mitochondrial function in cancer cells through ROS-MMP coupling modulation rather than ROS overproduction. These findings delineate both the shared and ligand-specific effects of different G4 ligands, reinforce mitochondria as cancer-selective vulnerabilities, and suggest that even through G4 structures represent a broad cellular target, G4-directed intervention can achieve a safer and more precise therapeutic strategy compared with conventional DNA-damaging agents such as cisplatin.

### Limitations of the study

Several limitations should be acknowledged. First, we evaluated a narrow panel of representative ligands (360 A, Phen-DC3, and Pt-ttpy), and broader coverage of structural diversity will be needed to generalize the classification of ROS-amplifying versus ROS-independent mitochondrial G4-targeting phenotypes. Second, while the EDL21 data supports G4-dependence, direct demonstration of ligand-mtG4 engagement in cells remains to be established. Third, our conclusions are based primarily on the *in vitro* high-content single-cell imaging in HeLa and primary fibroblasts, complemented by FACS and viability assays; *in vivo* validation in tumor models and clinically relevant systems will be important to assess translational relevance. Finally, the precise molecular basis of the 360 A-mediated ROS suppression, whether driven by altered electron transport, adaptive redox rewiring, antioxidant regulation, or mtG4-associated changes in mitochondrial gene expression, remains to be dissected and will be the focus of our future studies.

## Resource availability

### Lead contact

Further information and requests regarding resources and analyses should be directed to and will be fulfilled by the lead contact, Jia Tao (tao.jia86@outlook.com).

### Materials availability

This study did not generate new unique reagents.

### Data and code availability

• All original code is available in this paper’s supplemental information.
• 1H NMR and HR-MS of EDL21 data have been deposited at Biological Magnetic Resonance DataBank (BMRB) as accession number bmrbig144, and are publicly available as of the date of publication.
• Any additional information required to reanalyze the data reported in this article is available from the lead contact upon request.

## Acknowledgments

J.T. profoundly thanks Dr. Yu Luo from Institut Curie for a long tradition of insightful discussion with invaluable comments and suggestions on the project of investigating G4 ligands’ potential effects in modulating mitochondrial homeostasis, as well as critical review of this work. J.J. also deeply acknowledges Prof. Chengjian Zhao from the State Key Laboratory of Biotherapeutics for his selfless advice and support, which enabled our access to the National Facility for Translational Medicine (Sichuan) instruments and equipment. We also thank the College of Life Sciences at Sichuan University and Nikon Corporation for providing our access to the high-content microscopy platform, and Mr Qiang Zhang, Mr Shi Junyang, Miss Qian Zhu, and Mr. Jie Lin, the person in charge of the microscopy facilities, for their great technical support.

Funding: This research was supported by Central Guidance for Local Science and Technology Development Fund Project (202502ZYDF044), Health Commission of Sichuan Province Medical Science and Technology Program (25CXTD30), 10.13039/100018477Startup Foundation for Recruited High-level Talents of Mianyang Central Hospital (2025RCYJ-010), 10.13039/501100004829Science and Technology Department of Sichuan Province (2023NSFSC0130), and (2023NSFSC1992). The open-access publication fee was covered by Mianyang Central Hospital (2019FH02). All of the above funding sources are from China.

## Author contributions

T.J. conceived the study. X.Z., F.L., S.Z., Y.Q., Q.W., D.L., Q.C., X.G., J.D., Q.Q., and L.L. performed experiments and analyzed data. T.J. supervised the project. Q.L., X.Z., Z.S., A.G., T.J., S.B., and L.L. wrote and revised the manuscript. All authors approved the final version.

## Declaration of interests

The authors declare that they have no competing interests.

## STAR★Methods

### Key resources table

**Table:** 

REAGENT or RESOURCE	SOURCE	IDENTIFIER
**Antibodies**		

Anti-MT-CO1 antibody	ABclonal	Cat# A17889; RRID:AB_2861744
Anti-MT-ND6 antibody	ABclonal	Cat# A17991; RRID: AB_2861788
Anti-TFAM antibody	Cell Signaling Technology	Cat# 8076; RRID: AB_10949110
Anti-GAPDH antibody	Abcam	Cat# ab9485; RRID: AB_307275
HRP Goat Anti-Rabbit IgG(H + L)	ABclonal	AS014; RRID: AB_2769854
HRP-conjugated Goat Anti-Mouse IgG(H + L)	Proteintech	SA00001-1; RRID: AB_2722565

**Chemicals, peptides, and recombinant proteins**		

Pt-ttpy	Merck Sigma	Cat# SML2556
Cisplatin	West China Medical Center	N/A
360 A iodide	MedChemExpress	Cat# HY-15595 A
Phen-DC3 trifluoromethanesulfonate	MedChemExpress	Cat# HY-15594 A
EDL21	This paper	N/A
Hoechst 33342	MedChemExpress	Cat# HY-15559
TMRE	MedChemExpress	Cat# HY-D0985A
JC-1	MedChemExpress	Cat# HY-15534

**Critical commercial assays**		

ROS Assay Kit (Highly Sensitive DCFH-DA)	DOJINDO	Cat# R252
mtSOX Deep Red Mitochondrial Superoxide Detection	DOJINDO	Cat# MT14
CCK-8 assay	Sigma-Aldrich	Cat# 96992
Apoptosis Detection Kit (Annexin V-FITC/PI)	Yeasen	Cat# 40302ES60
PowerUp SYBR Master Mix	Applied Biosystems	Cat# A25742

**Deposited data**		

CellProfiler-extracted feature database in HeLa cells	This paper	Data S2
CellProfiler-extracted feature database in HPFC cells	This paper	Data S3
1H NMR and HR-MS of EDL21 data	This paper	[BMRbig]: [bmrbig144] (BMRbig: 1H NMR-HR-MS - EDL21)

**Experimental models: Cell lines**		

Human: HeLa cervical adenocarcinoma cells	Meisen CTCC	Cat# CTCC-001-0006; RRID:CVCL_0030
Human: NCI-H226 lung squamous carcinoma cells	Meisen CTCC	Cat# CTCC-400-0169; RRID:CVCL_1544
Human: A2780 ovarian carcinoma cells	Meisen CTCC	Cat# CTCC-003-0011; RRID:CVCL_0134
Human: primary fibroblast cells (HPFCs)	This paper	N/A

**Oligonucleotides**		

β-Actin fw: TCACCCACACTGTGCCCATCTACGA	This paper	N/A
β-Actin rv: CAGCGGAACCGCTCATTGCCAATGG	This paper	N/A
12 S fw: GCTCGCCAGAACACTACGAG	This paper	N/A
12 S rv: CAGGGTTTGCTGAAGATGGC	This paper	N/A
COX1 fw: AATAGGAGCTGTATTTGCCAT	This paper	N/A
COX1 rv: AGAAAGTTAGATTTACGCCGAT	This paper	N/A
ND6 fw: ATATACTACAGCGATGGCTA	This paper	N/A
ND6 rv: AATCCTACCTCCATCGCTA	This paper	N/A

**Software and algorithms**		

GraphPad Prism	GraphPad Software, LLC	V9 https://www.graphpad.com/
Fiji ImageJ software	ImageJ.org	https://doi.org/10.1038/nmeth.2019
CellProfiler	cellprofiler.org	V.4.2.8 https://cellprofiler.org/
R	R Foundation for Statistical Computing	https://www.r-project.org/
RStudio	Posit	Download RStudio - Posit
FlowJo	BD Biosciences	https://www.flowjo.com/
Custom Fiji macros for single-cell quantification	This paper	Data S1
Custom R scripts for Heatmap	This paper	Data S4
Custon R scripts for PCA	This Paper	Data S5

**Other**		

96-well glass-bottom plates for high-content imaging	Corning	Cat# 4580
Nikon ECLIPSE Ji digital imaging microscope	Nikon	Nikon ECLIPSE JI

### Experimental model and study participant details

#### Cell line

Human cervix cancer cell line HeLa (catalog no. CTCC-001-0006, Meisen CTCC), Human lung cancer cell line H226 (catalog no. CTCC-400-0169, Meisen CTCC), Human ovarian cancer cell line A2780 (catalog no. CTCC-003-0011, Meisen CTCC), Human primary fibroblast cells (HPFC) were sorted by CD106 antibody (#130-122-339, Miltenyi Biotec) with the kit of Dynabeads FlowComp Flexi (#11061D, ThermoFisher) following our previous protocols.^16^ HeLa and HPFC were cultured in complete DMEM medium supplemented with 10% fetal bovine serum (FBS, catalog no. Z7185FBS-500, ZETA life) and 100 U/mL penicillin +100 μg/mL streptomycin (catalog no. Gibco-15,140,122, ThermoFisher, Gibco). H226 was cultured with RPMI 1640 medium with 10% FBS with penicillin and streptomycin. Cells were incubated under a 5% CO2 humidified incubator at 37°C. When it reached 80–90% fusion, cells were digested with 0.25% trypsin/0.91 mM EDTA (catalog no. Gibco 2,520,072, ThermoFisher), then collected for indicated experiments.

#### Cell line authentication and mycoplasma testing

All cancer cell lines utilized in this study underwent rigorous authentication, in the beginning, through STR profiling and mycoplasma testing to ensure their identity and contamination-free status. During the experimental period, mycoplasma testing was performed around every three months. Only cell lines that tested negative for mycoplasma were used for subsequent research experiments.

### Method details

#### Compounds

Pt-ttpy was obtained from Merck Sigma (catalog no. SML2556). Cisplatin was kindly provided by the West China Medical Center. 360 A iodide (360 A) was purchased from MedChemExpress (MCE, catalog no. HY-15595 A) and Phen-DC3 Trifluoromethanesulfonate (Phen-DC3) was provided by MCE (catalog no. HY-15594 A).

#### Synthesis of EDL21

The compound EDL21 was synthesized via a two-step procedure (Scheme S1) following the original report.^15^ The identity and purity of EDL21 were unequivocally confirmed by ^1^H NMR and high-resolution mass spectrometry (HR-MS). Detailed synthetic protocols and characterization data are provided in the Supporting datasheet of chemical part. For all experiments, EDL21 was prepared at 10 mM stock solution in DMSO and stored at 4°C. The solvent for EDL21 and all other ligands was DMSO, except for cisplatin which has been dissolved in water at 17 mM water, with corresponding DMSO control solutions included. The final working concentration of DMSO in all assays was maintained at 0.1% (v/v).

#### Cell plating and compounds treatment

HeLa and HPFC cells were seeded into 96-well glass-bottom plates (designed for high-content screening, Corning, catalog no. 4580) at a density of 3 × 10^3^ cells per well. For each plate, both cell types were processed in parallel, with six treatment groups defined: Control, EDL21, Pt-ttpy, 360 A, Phen-DC3, and Cisplatin. Each treatment was performed in at least duplicate wells per plate, and peripheral wells were filled with Hanks’ balanced salt solution (HBSS) to minimize edge effects. At least five independent experiments were performed to ensure reproducibility.

Cells were treated with the selected compounds at two concentrations based on previous literature: a low concentration of 1 μM and a high concentration of 10 μM. After 24 h of cell adhesion, the indicated ligands were added for an additional 24 h of incubation. Subsequently, live-cell dyes for mitochondrial function were applied, and cells were subjected to Mito-HiCP for following quantitative multiparametric analysis.

#### Cell painting and staining

The effects of G4 ligands or Cisplatin on total ROS (ROS Assay Kit - Highly Sensitive DCFH-DA, #R252, DOJINDO), mitochondrial ROS (mtSOX Deep Red-Mitochondrial Superoxide Detection, #MT14, DOJINDO), and mitochondrial membrane potential (ΔΨm) (TMRE, HY-D0985A) were evaluated following the manufacturer’s protocols. Hoechst 33342 (catalog no. HY-(15559) was used for nuclear staining. Indicated fluorescent probes were employed for detecting various cellular components (see Table S1). Intracellular ROS and mitochondrial superoxide were detected using the respective working solutions prepared according to the manufacturers’ instructions. Briefly, the mtSOX Deep Red probe was reconstituted in 10 μL DMSO to generate a stock solution, which was stored at −20°C and subsequently diluted 1:1000 in HBSS (Gibco, catalog no. C14175500 BT) before use. The DCFH-DA dye was diluted 1:1000 in 1× Loading Buffer, which was prepared by 10-fold dilution of the 10× stock with ultrapure water (Invitrogen, catalog no. 10977015). The two working solutions were combined at a 1:1 ratio, supplemented with Hoechst 33342 (final concentration: 10 μg/mL) and TMRE (final concentration: 1 μM). Following 30 min of incubation at 37°C in the dark, cells were washed and imaged in 200 μL of fresh HBSS.

#### Imaging and data mining, analysis and visualization

Fluorescent images were captured using a Nikon ECLIPSE Ji Digital Imaging Microscope with a 40× objective, utilizing laser-based autofocus and a 4× Panorama Scan. For each well, four sites were imaged, capturing Bright Field (BF) and four fluorescence channels. The combined analysis pipeline using Fiji, CellProfiler and R is detailed in the supplementary document.

#### Cell proliferation assays

Cell proliferation was assessed using CCK8 (Sigma-Aldrich, #(96992) assay For the CCK8 assay, 3 × 10^3^ tumor cells were seeded in 96-well plates with 200 μL culture medium and allowed to adhere overnight. Then cells were treated with Pt-ttpy, 360 A, Phen-DC3, cisplatin, EDL21 at the 1 μM, 5 μM and 10 μM, for 24 h. Following treatment, cells were incubated with CCK8 in a serum-containing medium for 1 h before measuring absorbance at OD450 nm using a plate reader (Thermo Fish Scientific Multiskan FC Microplate Photometer).

#### Total ROS and mitochondrial membrane potential detection by FACS

Flow cytometry to confirm the levels of ROS and MMP changes in HeLa cells post indicated G4 ligands and control compounds treatment. The cells were treated with 10 μM Pt-ttpy, 360 A, Phen-DC3, cisplatin, EDL21 for 24 h, harvested, and washed with PBS. The cells were then incubated with a ROS probe (DOJINDO, catalog no. #R252)/mtROS (DOJINDO, catalog no. #MT14) mixed dye solution or JC-1 (MCE, catalog no. HY-(15534) dye solution at 37°C for 30 min. After washing with HBSS, the samples were loaded for analysis using a Beckman coulter flow cytometer. And data was analyzed using FlowJo software.

#### Western blotting

HeLa cells were seeded in 6-well plates at a density of 0.1–0.2 × 10^6^/well and cultured in complete medium for 24 h. The medium was then replaced with fresh medium containing the indicated concentrations of 360 A (10 μM or 20 μM). After treatment for 24 or 72 h, total proteins were extracted using RIPA Lysis Buffer (1×, Cell Signaling Technology) supplemented with 1× EDTA-free Protease Inhibitor Cocktail (Roche) immediately before use.

Protein concentrations were determined, and approximately 20 μg of total protein per sample was separated by electrophoresis on Precast Protein Plus Gel, 4–20% (Yesen, catalog no.40302ES60, China). Subsequently, proteins were transferred onto PVDF membranes (Immobilon-PSQ, Merck Millipore, catalog no. ISEQ00010) using a Trans-Blot Turbo Transfer System (Bio-Rad) at a constant voltage of 90 V for 90 min.

The membranes were blocked and then incubated overnight at 4°C with the following primary antibodies diluted in 5% BSA: anti-MT-CO1 (1:1000, ABClonal, A17889), anti-MT-ND6 (1:1000, ABClonal, A17991), anti-TFAM (1:1000, Cell Signaling Technology, #8076), and anti-GAPDH (1:8000, Abcam, ab9485). After washing, membranes were incubated with the corresponding horseradish peroxidase (HRP)-conjugated secondary antibodies: either HRP Goat Anti-Rabbit IgG(H + L) (1:5000, ABclonal, AS014) or HRP-conjugated Affinipure Goat Anti-Mouse IgG(H + L) (1:5000, Proteintech, SA00001-1), at room temperature for 2 h.

Protein bands were visualized using a chemiluminescence detection system (Bio-Rad). Signal intensities of the target bands were quantified using ImageJ software.

#### Apoptosis assay

To evaluate the effect of G4 Ligands on apoptosis, the cells were treated with indicated con. of Pt-ttpy, 360 A, Phen-DC3, cisplatin, EDL21 for 96 h, harvested, and washed with PBS. Then cells were harvested, resuspended in binding buffer, and stained with 5 μL Annexin V-FITC and 10 μL of Propidium Iodide using Apoptosis Detection Kit (Yesen, catalog no.40302ES60, China) for 15 min at room temperature. After, indicated samples were loaded for analysis using a Beckman coulter flow cytometer. And data was analyzed using FlowJo software.

#### Fluorescent quantitative PCR and fluorescent quantitative RT-PCR

SYBR probes (POWRUP SYBR MASTER MIX, catalog no. A25742, applied biosystems by Thermo Fisher Scientific) were used in a 25 μL system. Reaction conditions were following the manufacturer’s protocol.

According to the instructions provided by Nanjing Vazyme Biotech (Jiangsu, China), qPCR and RT-qPCR experiments were conducted. DNA or RNA quantity was determined by NanoDrop (Thermo Fisher). The primer sequences were designed and provided by Chengdu Youkangjianxing Biotechnology Co., Ltd. (Chengdu, Sichuan, China) and can be seen in Table S2. using a QuantStudio 5 real-time PCR system by conventional settings (Applied Biosystems).

The amplification conditions were as follows: 95°C for 30 s, 95°C for 5 s, and 60°C for 34 s, for a total of 45 cycles. The data were analyzed using the ΔΔCT method, and β-Actin serving as the internal reference gene. The formula is as follows: ΔΔCt = ΔCt experimental group - ΔCt control group, where ΔCt = Ct (target gene) - Ct (reference gene).

### Quantification and statistical analysis

Details of the statistical methods, definitions of significance, and result summaries are detailed in indicated figure legends and the results section. All error bars represent the SEM. Statistical significance was analyzed using Unpaired t tests or One-way ANOVA, as appropriate. Correlation analyses were performed using Pearson correlation coefficients, and differences between correlation coefficients were assessed using Fisher’s z-tests. All statistical analyses were conducted using GraphPad Prism. A *p* value <0.05 was considered statistically significant (∗*p* < 0.05, ∗∗*p* < 0.01, ∗∗∗*p* < 0.001).

## Supplemental information

Document S1. Figures S1–S15, Scheme S1, Tables S1 and S2 and Methods S1

Table S3. Correlation of mtROS and MMP in HPFC and HeLa, related to Figure 5

Data S1. Custom Fiji macros for single-cell quantification, related to Figure 1and STAR Methods

Data S2. CellProfiler-extracted feature database in HeLa cells, related to Figure 3

Data S3. CellProfiler-extracted feature database in HPFC cells, related to Figure 3

Data S4. Heatmap Rstudio macro, related to STAR Methods

Data S5. PCA Rstudio macro, related to STAR Methods

Data S6. Full unedited wastern blots
