# Supplementary material for: Do G4 ligands induce mitochondrial dysfunction without ROS induction
Source: iScience. 2026 May 22;29(6):116036. doi: 10.1016/j.isci.2026.116036 (PMC13224012; doi:10.1016/j.isci.2026.116036)
Supplement: Document S1. Figures S1–S15, Scheme S1, Tables S1 and S2 and Methods S1 [file mmc1.pdf]

iScience, Volume *29*

Supplemental information

Do G4 ligands induce mitochondrial dysfunction without ROS induction

Xinru Zhang, Fei Li, Siyi Zeng, Dongyin Lian, Qi Cheng, Lin Li, Xin Gao, Qiantao Wang, Yumei Qin, Junrong Du, Qiang Li, Zhenlei Song, Qingrong Qi, Anton Granzhan, Sophie Bombard, and Tao Jia (贾涛)

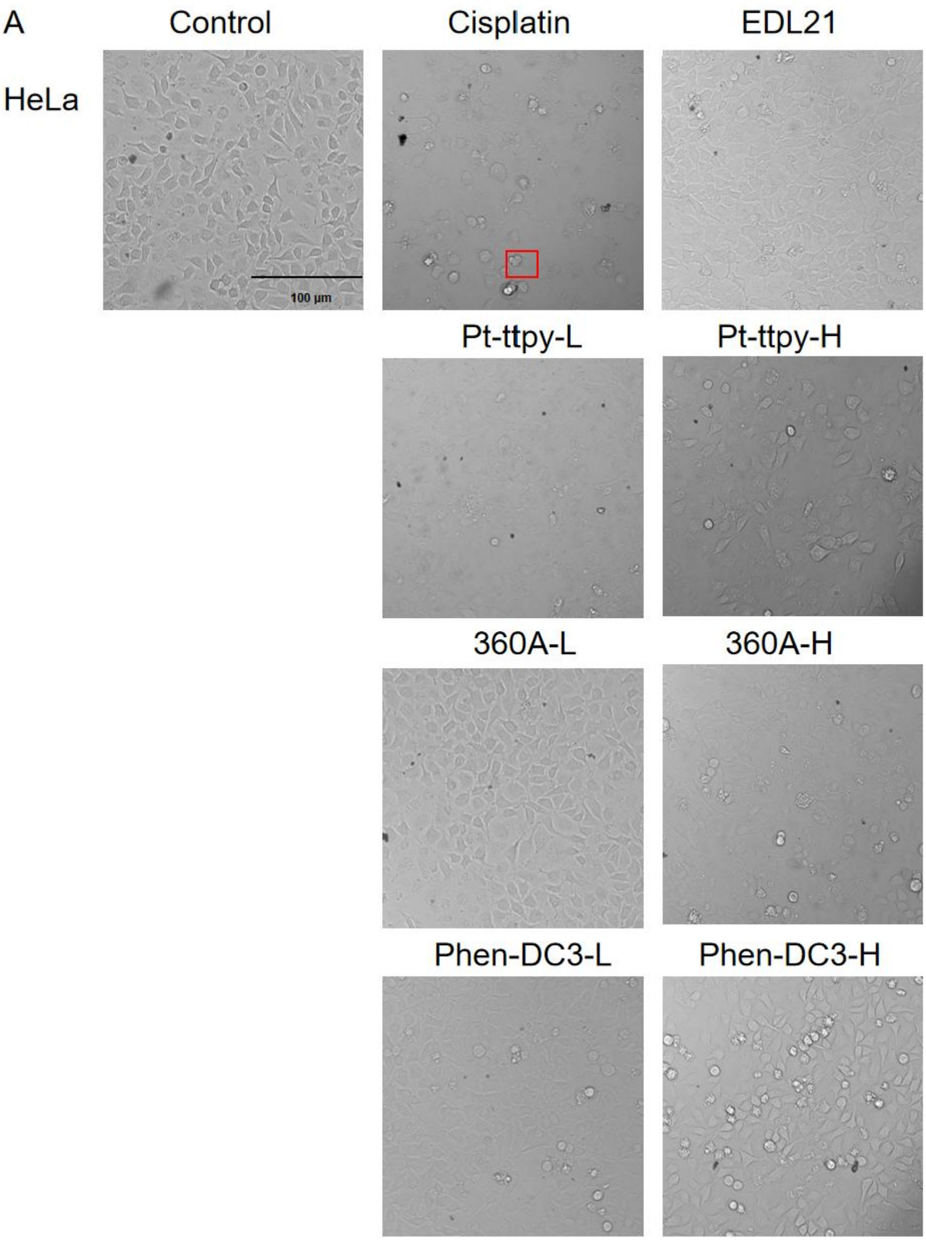
A
Control
Cisplatin
EDL21
HeLa
100 µm
Pt-ttpy-L
Pt-ttpy-H
360A-L
360A-H
Phen-DC3-L
Phen-DC3-H

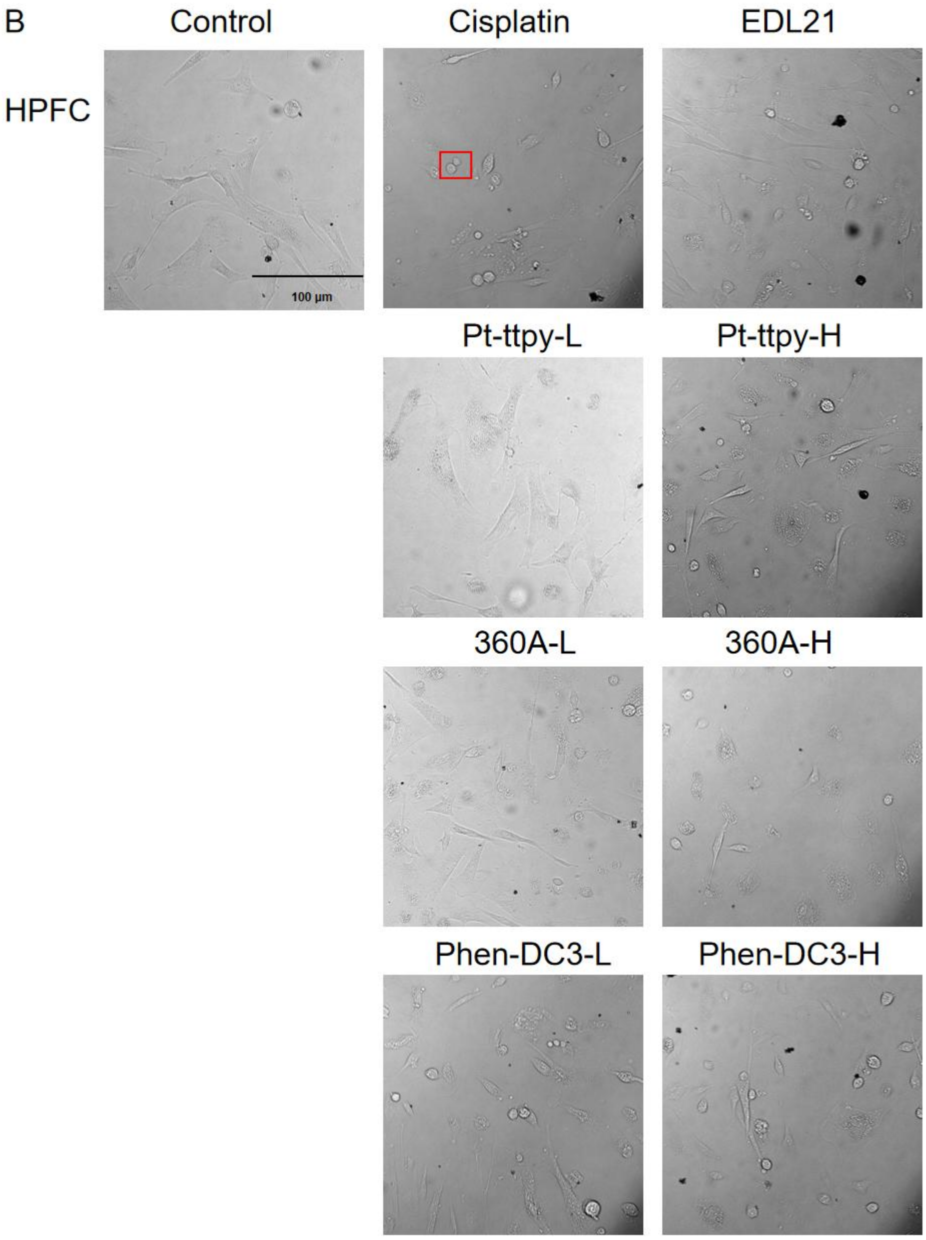

Figure S1. Bright-field images of HeLa (A) and HPFC (B) cells following treatment with G4 ligands, Cisplatin and EDL21. Related to Figure 2. (A) HeLa cells were exposed for 24 h to G4 ligands (Pt-ttpy, 360A, Phen-DC3) at low (**L**, 1 µM) and high (**H**, 10 µM) concentrations, as well as to cisplatin (10 µM) and EDL21 (10 µM). Images were acquired using a high-content microscope at 20× magnification. (B) HPFC cells were treated under the same conditions. Red boxes mark dead or floating cells. Scale bar = 100 µm.

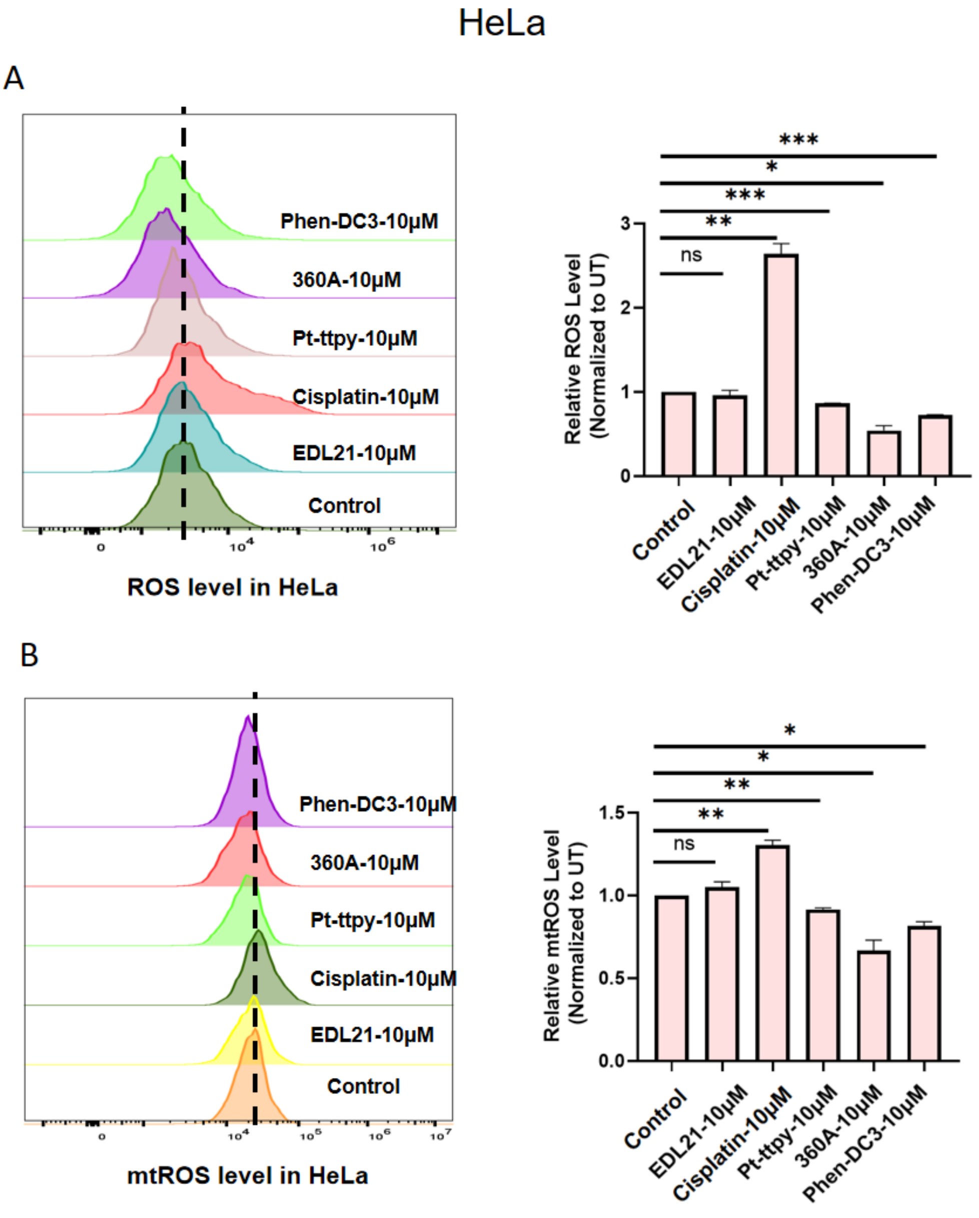

Figure S2. Flow cytometry analysis of ROS (A) and mtROS (B) in HeLa cells following indicated G4 ligands' treatment (10 μM) for 24 h. Related to Figure 2. Left panels display the measured parameters across different FACS channels, as shown in the figure. The right indicates the corresponding bar graphs, representing median of normalized fluorescence intensity. Data represents two independent experiments with the mean ± SEM. Data were analyzed using an unpaired t-test to evaluate the significance of differences between each treatment group and the control group. *$P < 0.05$, **$P < 0.01$, ***$P < 0.001$.

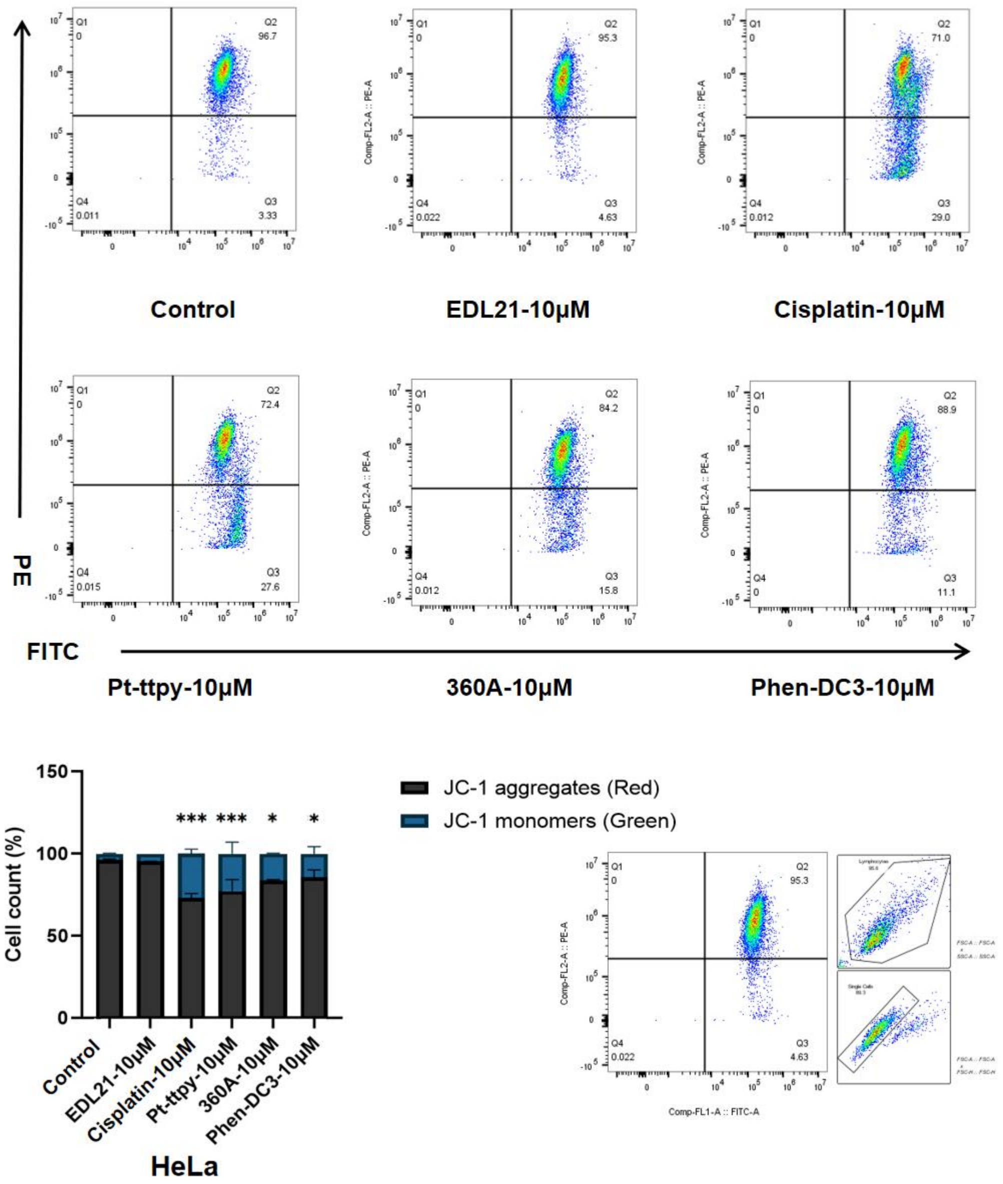

Figure S3. Flow cytometry analysis of JC-1 in HeLa cells treated with EDL21, cisplatin, Pt-ttpy, 360A and Phen-DC3 for 10 μM for 24 h. Related to Figure 2. Data were analyzed using an two way ANOVA to evaluate the significance of differences between each treatment group and the control group. *P < 0.05, **P < 0.01, ***P < 0.001.

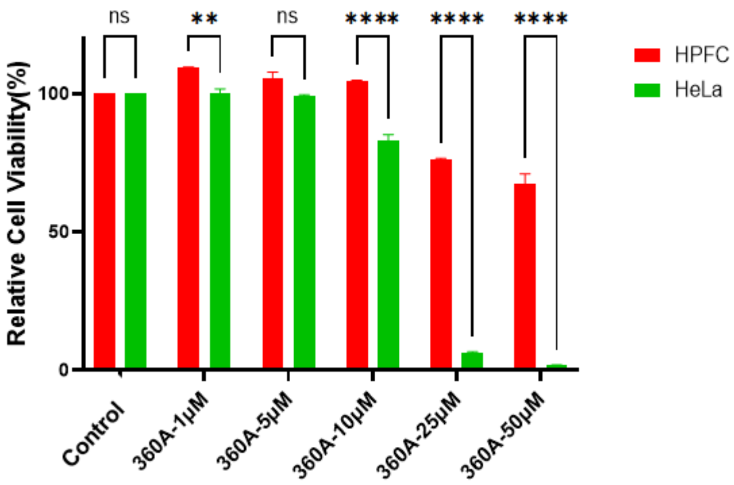

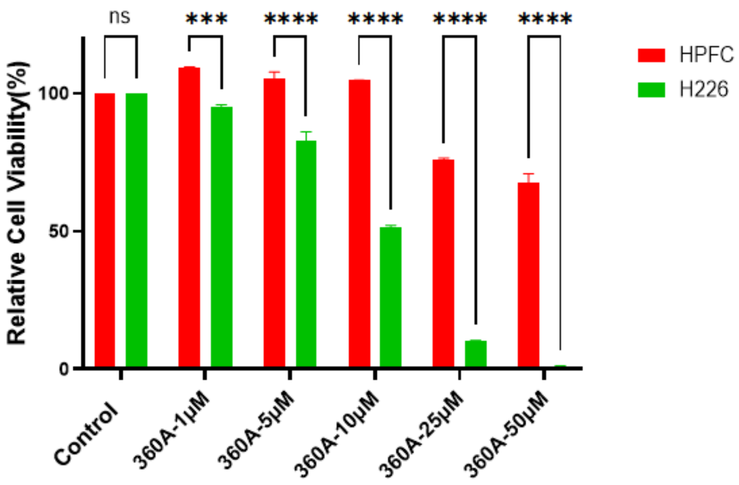

Figure S4. Cell Viability (CCK-8 assay) Responses of cancer cells HeLa, H226, and primary normal cells HPFC to G4 ligand 360A. Related to Figure 2. Data were analyzed using an unpaired t-test to evaluate the significance of differences between each treatment group and the control group. $*P < 0.05$, $**P < 0.01$, $***P < 0.001$. Data shown as mean ± SEM of three independent experiments.

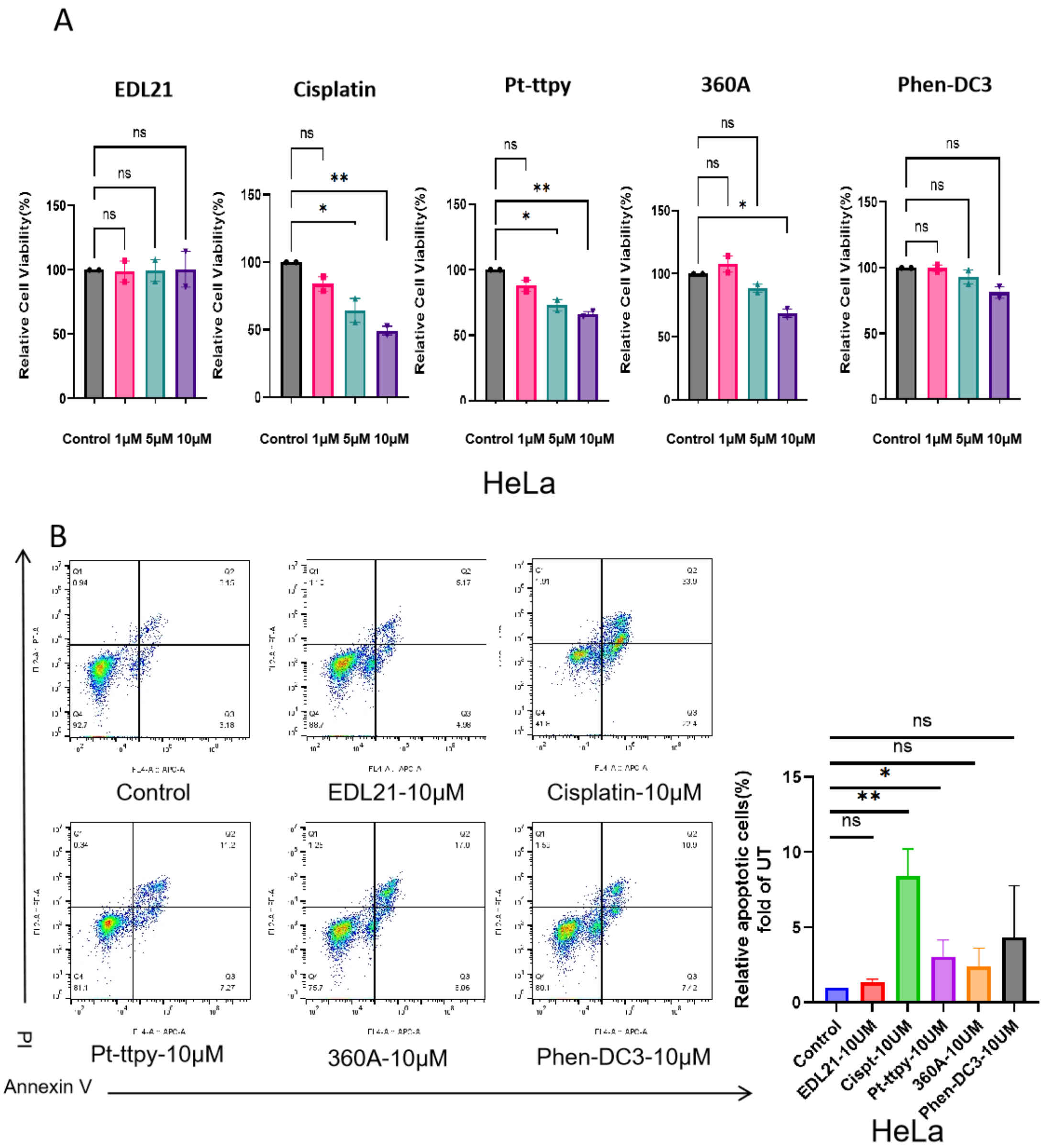

Figure S5. Cell viability and apoptosis (%) under treatment with G4 ligands and controls. Related to Figure 2. Data were analyzed using an unpaired t-test to evaluate the significance of differences between each treatment group and the control group. *P < 0.05, **P < 0.01. (A) CCK-8 assay. Data shown as mean ± SEM of two independent experiments. (B) apoptosis detection. Data shown as mean ± SEM of three independent experiments.

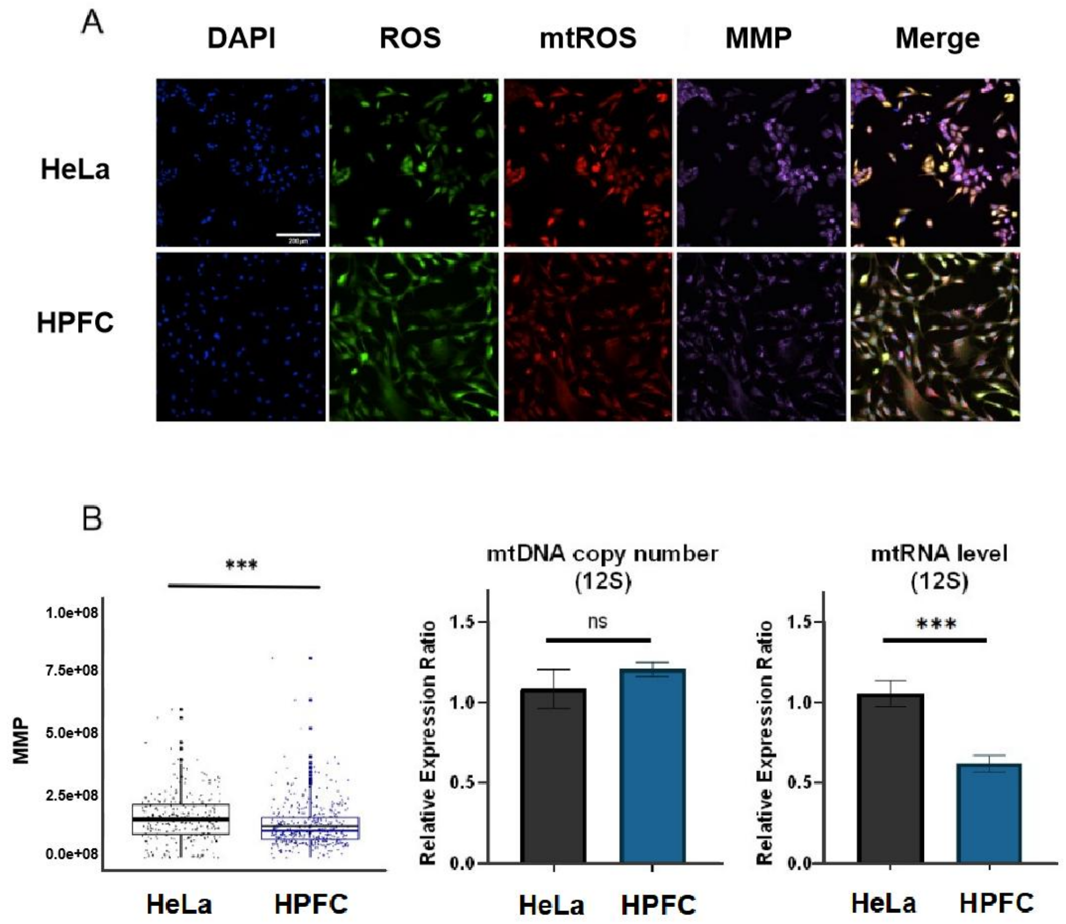

Figure S6. Comparative studies of Mitochondrial membrane potential, Mitochondrial DNA copy number, and Mitochondrial RNA levels between HeLa and HPFC cells. Related to Figure 2. (A) Indicated images with different staining for HeLa and HPFC cells. The images were retrieved from the high content microscope image collection, scale bar: 200 μm. DAPI: nucleus; ROS: general ROS staining; mtROS: mitochondrial specific ROS staining; Mito Activity: mitochondrial membrane potential (MMP). (B) Single-cell fluorescence quantification of Mito Activity in both HeLa and HPFC cells. Black lines represent the mean values. qPCR and RT-qPCR quantification of mtDNA and mtRNA levels in HeLa and HPFC cells. Data represents two independent experiments with the mean ± SEM. P values were calculated toward the HeLa. ***$P < 0.001$, unpaired t-Student test; ns: no significance.

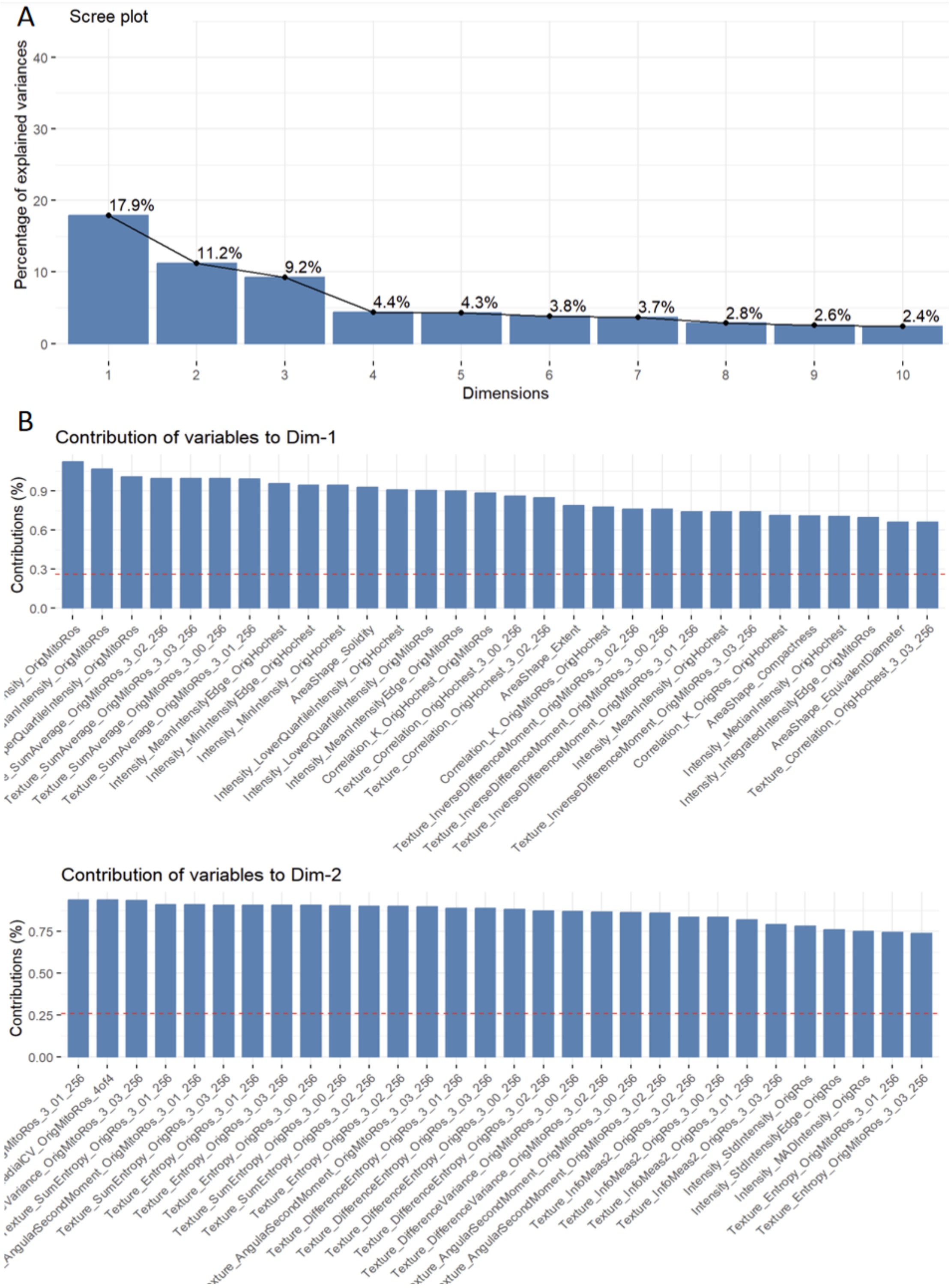

Figure S7. PCA features analysis. Related to Figure 3. (A) Scree plot: Show the variance analysis of each principal component. (B) Contribution plot of variables to PC1 and PC2, here we show the top 30.

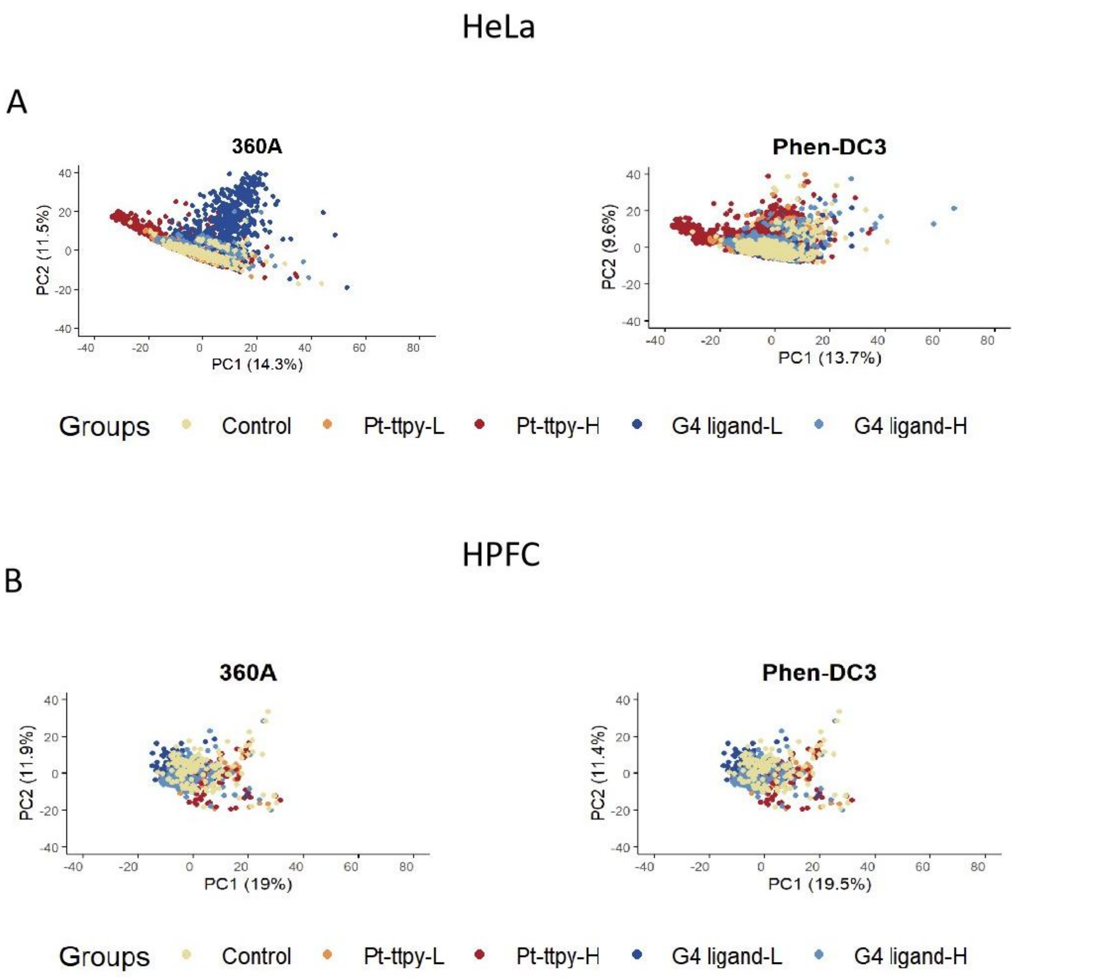

Figure S8. PCA analysis. Phenotypic feature analysis of different G4 ligands (360A, Phen-DC3) compared to Pt-ttpy at low (L, 1 μM)) and high (H, 10 μM)) con. relative to the control group in HeLa (A) and HPFC (B) cells. Related to Figure 3. PCA plots show dimensionality reduction of CellProfiler features extracted from each treatment group, displaying sample distribution in two-dimensional space. (A, B) HeLa and HPFC cells were treated with low (1 μM) and high (10 μM) concentrations of 360A, Phen-DC3, and Pt-ttpy (24-hour treatment). Their sample distribution in PCA was compared with the control and Pt-ttpy groups, grouping as follows: control, Pt-ttpy low concentration, Pt-ttpy high concentration, and low concentration G4 ligand.

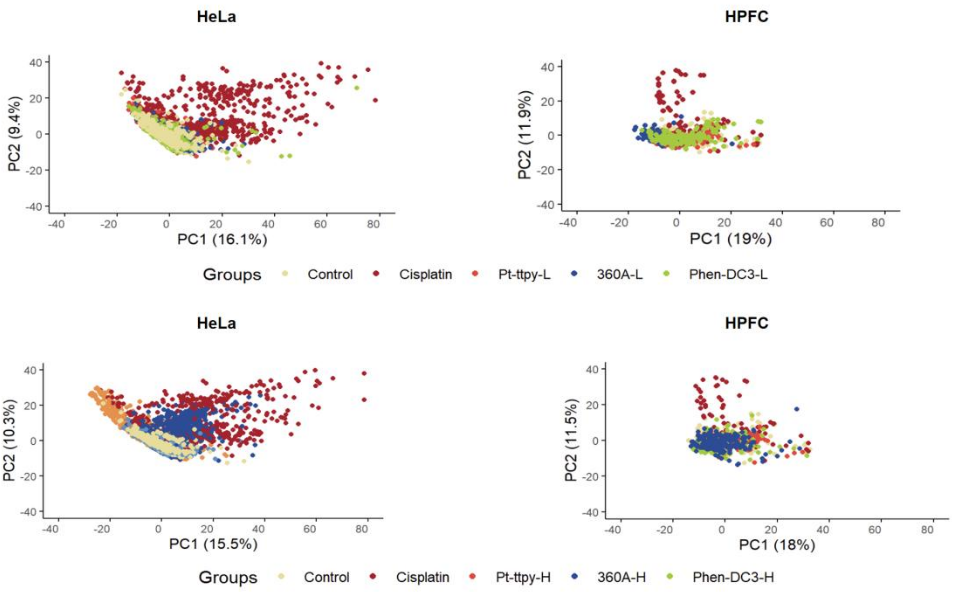

Figure S9. PCA plot show sample distributions of treatment groups compared with cisplatin and different G4 ligand at low (L, 1 μM) and high (H, 10 μM) concentrations. Related to Figure 3. HeLa and HPFC cells were treated with 360A, Phen-DC3, and Pt-ttpy at low (1 μM) and high (10 μM) concentrations, as well as cisplatin (10 μM) and EDL21 (10 μM) for 24h.

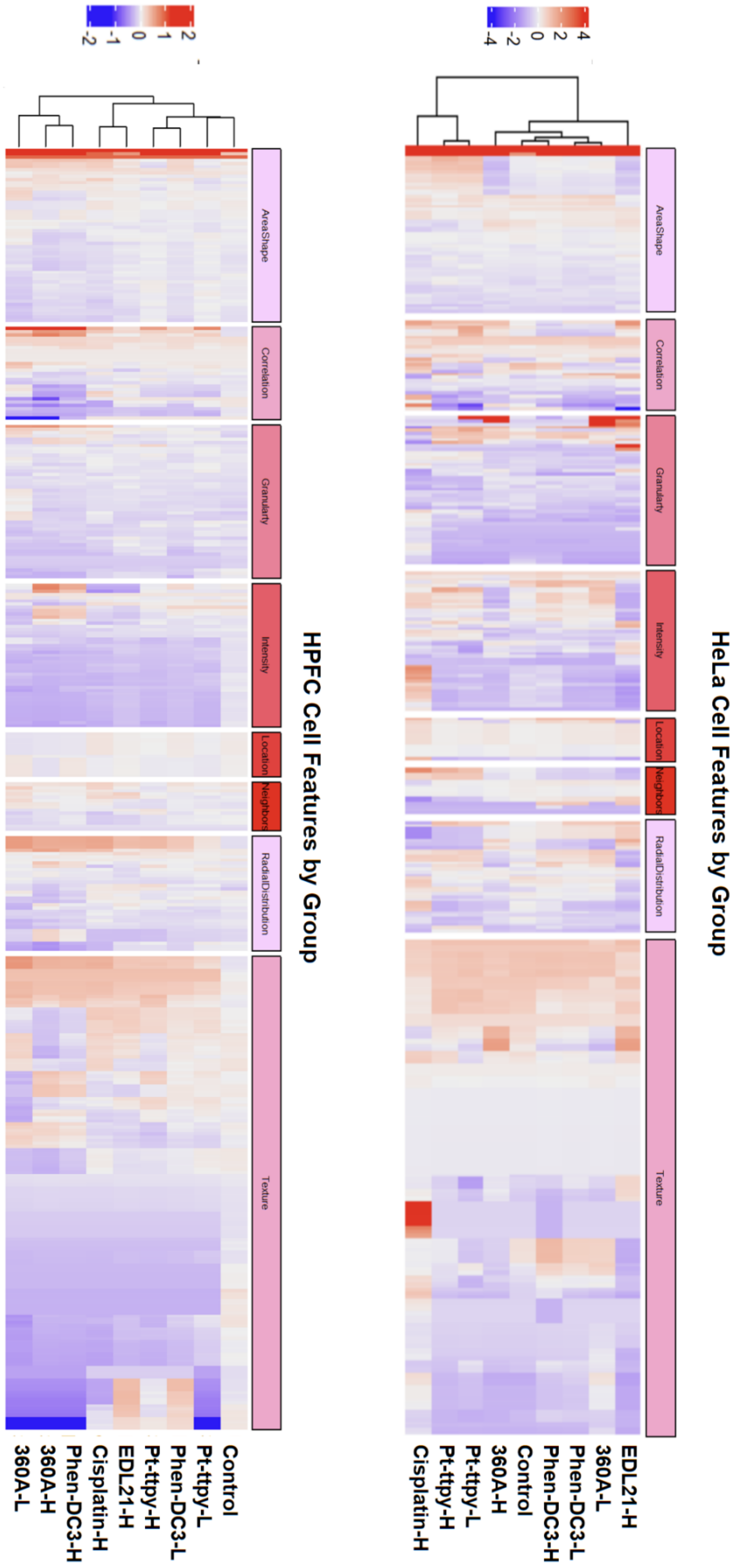

Figure S10. Heatmap clustering analysis of phenotypic features extracted by CellProfiler, comparing HeLa and HPFC cells across all treatment

groups. Related to Figure 3.

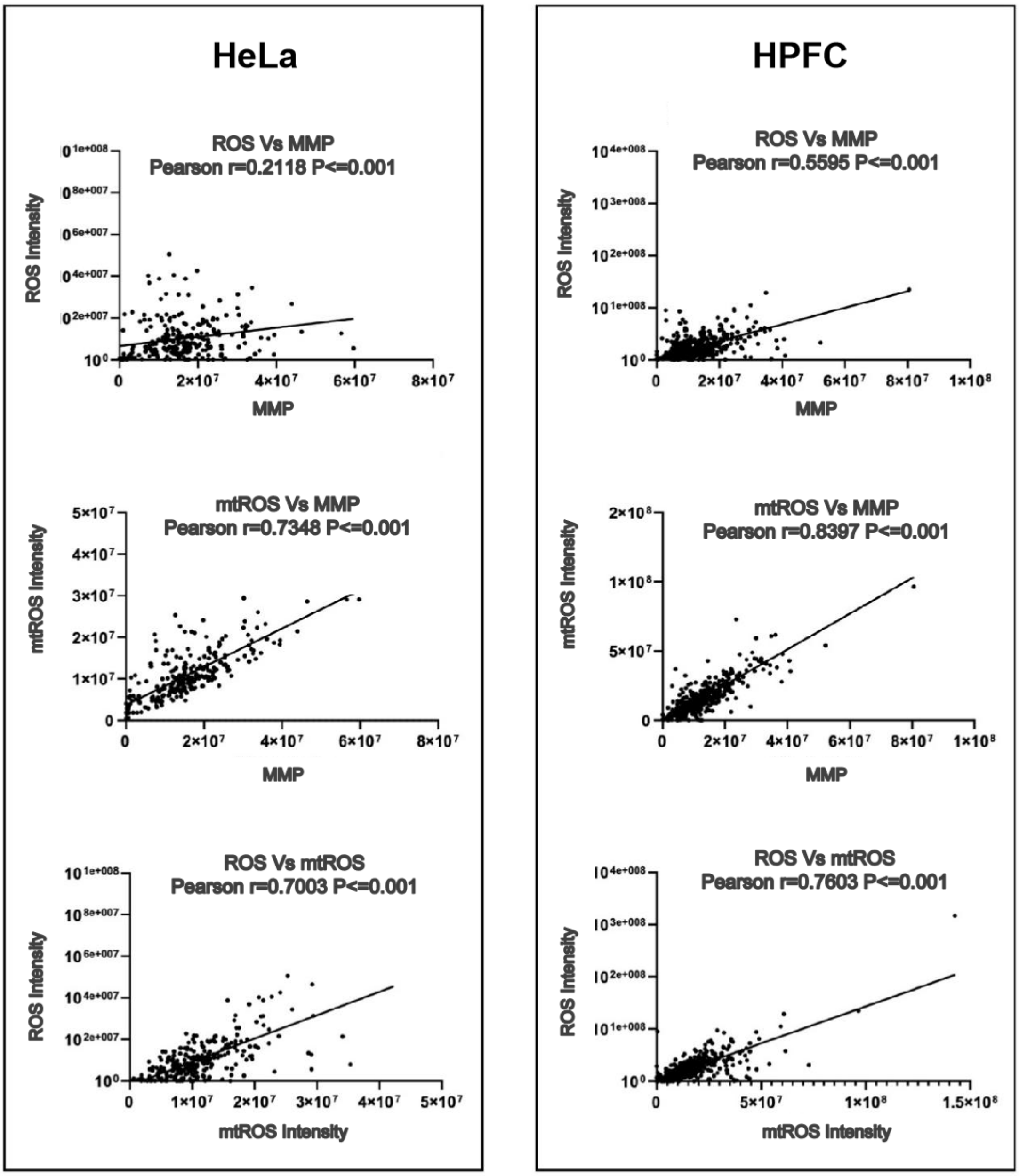

Figure S11. Correlation analysis between ROS levels and mitochondrial membrane potential (MMP) in control groups of normal (HPFC) and tumor (HeLa). Related to Figure 5. The Pearson r was calculated to assess correlation efficiency, the p-values were less than or equal to 0.001.

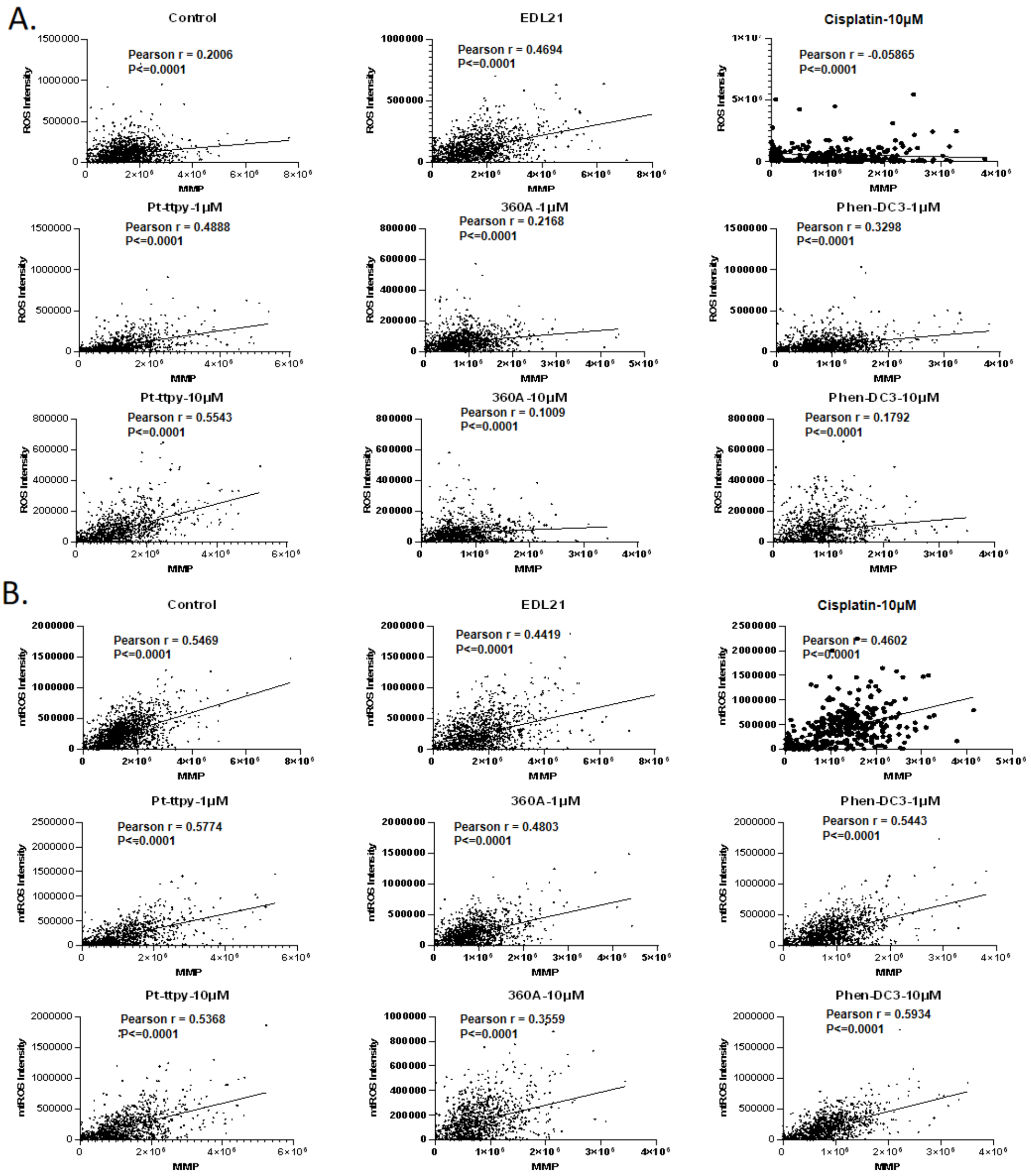

Figure S12. Correlation studies between ROS/mtROS and MMP in HeLa cells treated with different G4 ligands for 24 hours. Related to Figure 5. HeLa cells treated with 360A, Phen-DC3, Pt-ttpy at low (1 μM) and high (10 μM) concentrations, as well as Cisplatin (10 μM) and EDL21 (10 μM) for 24 h.The Pearson r was calculated to assess correlation efficiency, the p-values were less than or equal to 0.001.

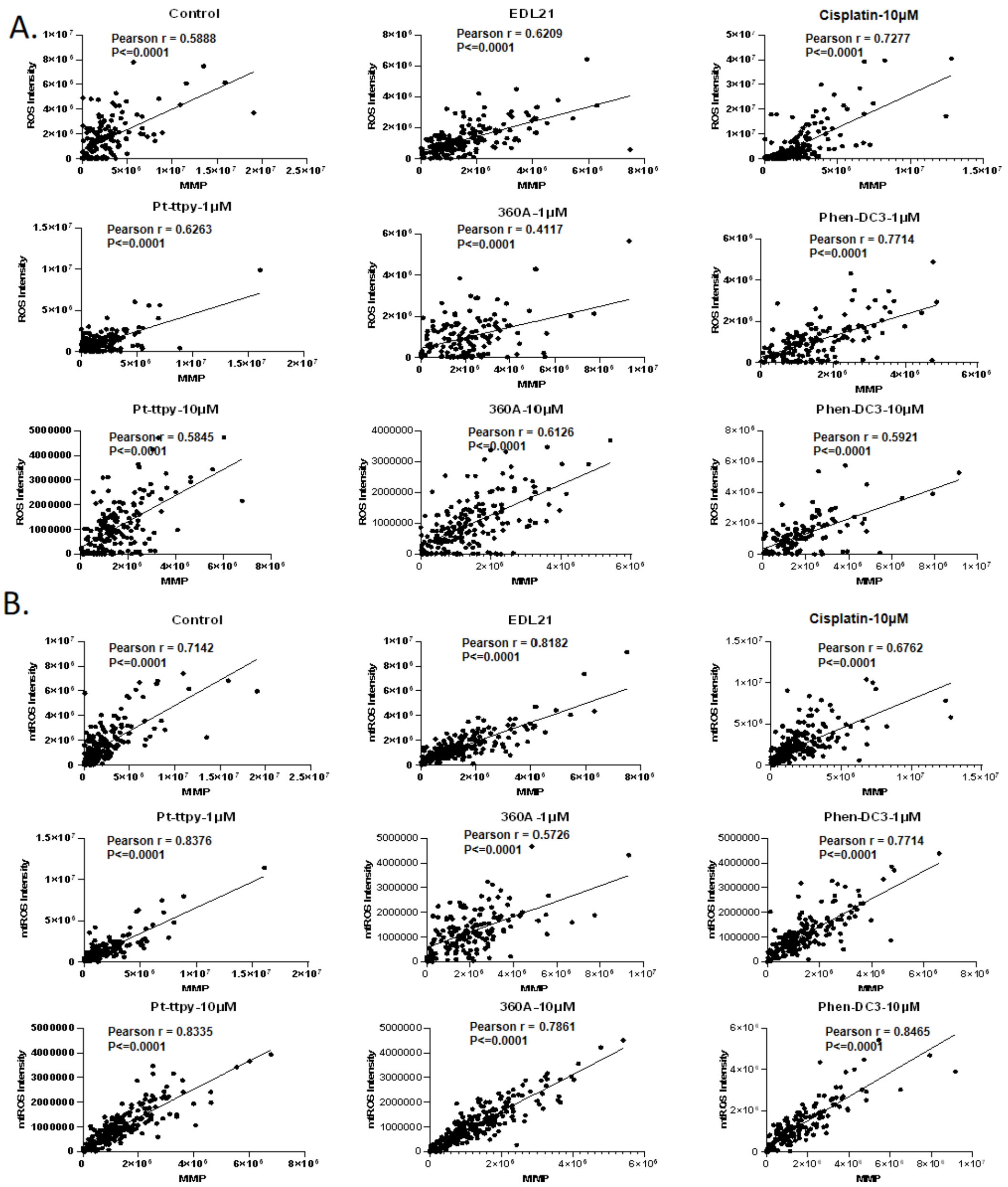

Figure S13. Correlation studies between ROS/mtROS and MMP in HPFC cells treated with different G4 ligands for 24 hours. Related to Figure 5. HPFC cells treated with 360A, Phen-DC3, Pt-ttpy at low (1 μM) and high (10 μM) concentrations, as well as Cisplatin (10 μM) and EDL21 (10 μM) for 24 h.The Pearson r was calculated to assess correlation efficiency, the p-values were less than or equal to 0.001.

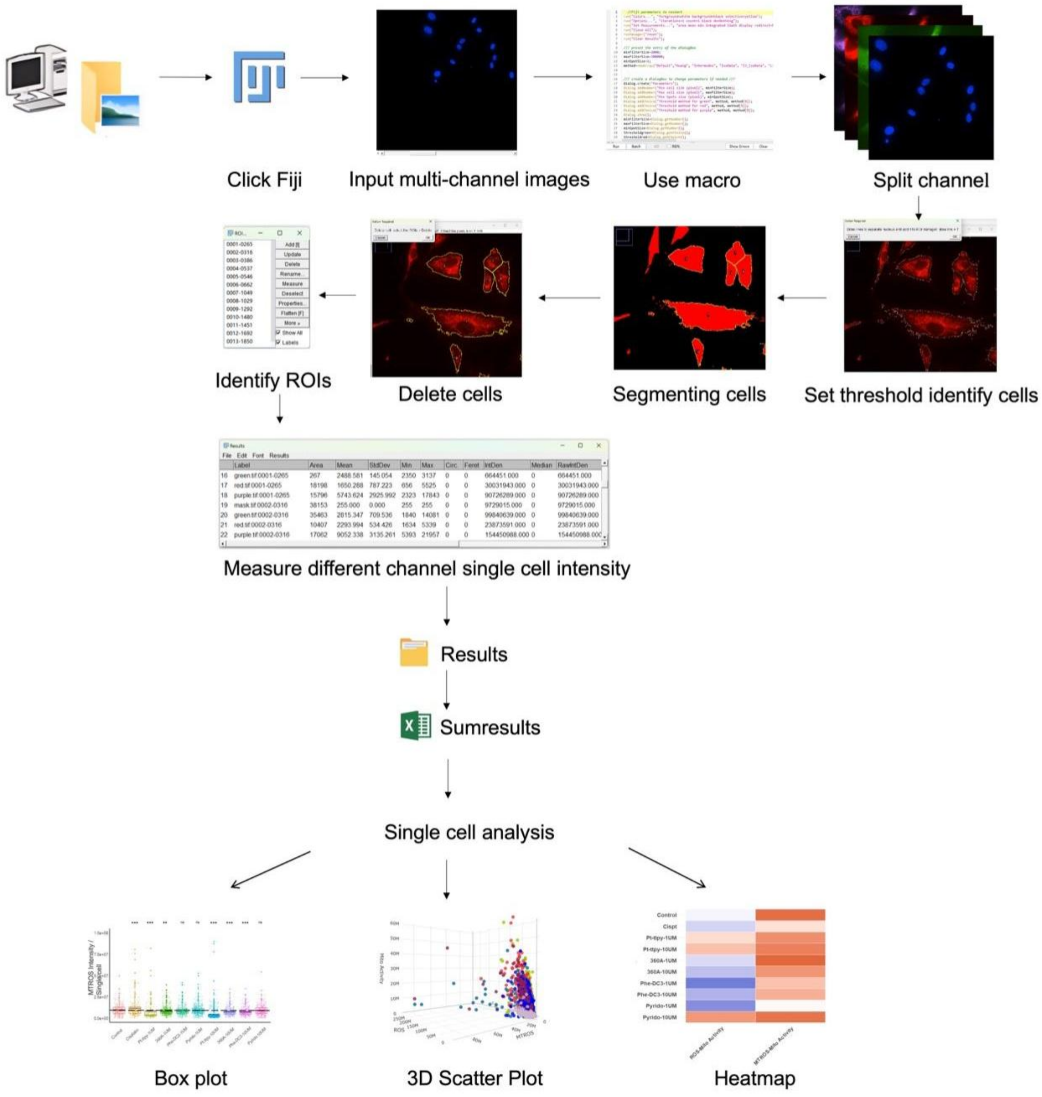

Figure S14. Fiji-based single cell analysis flowchart. Related to Figure 1.

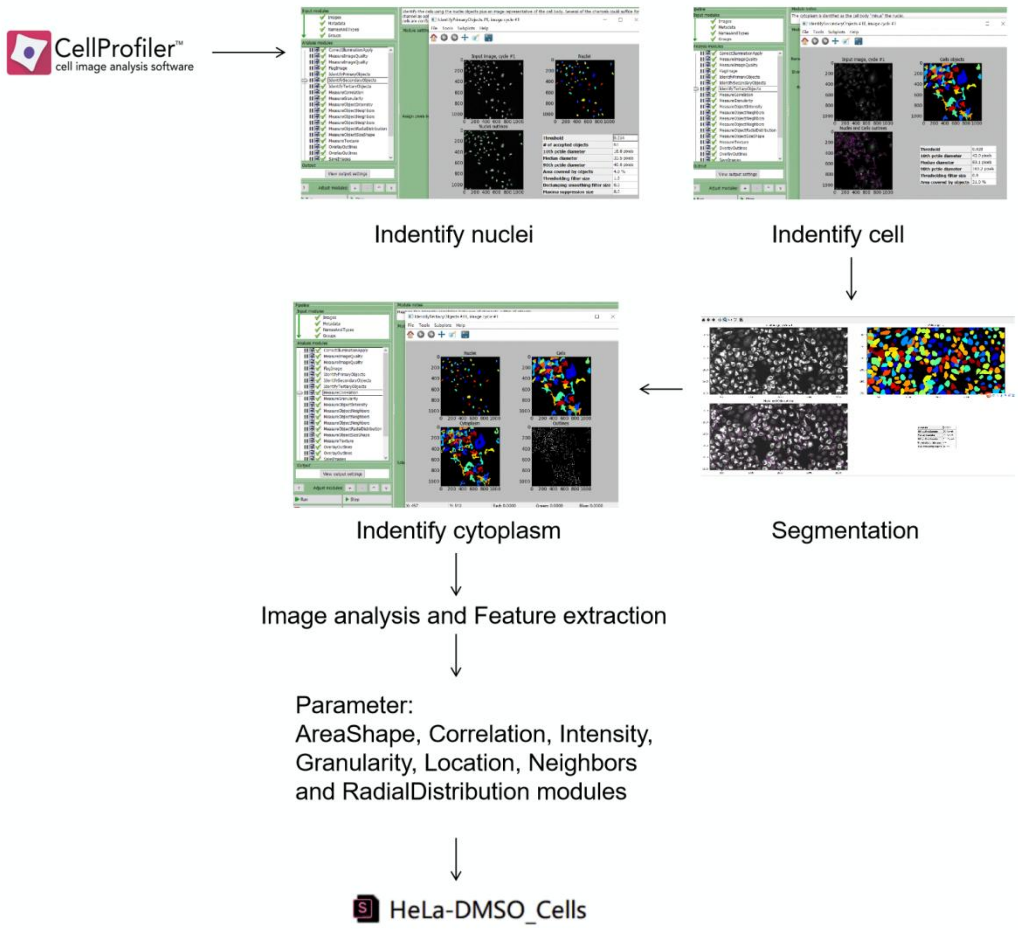

Figure S15. Cellprofiler segmentation overlays. Related to Figure 1.

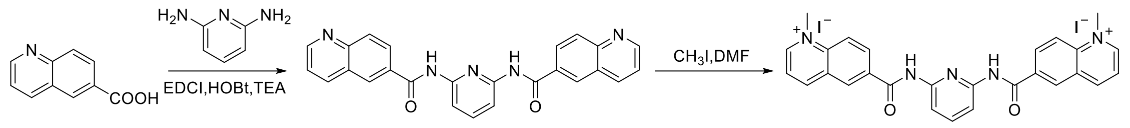

Scheme S1. The synthetic route for preparing EDL21.

EDL21-a[1]**:** EDCI (550 mg, 2.89 mmol, 3.1 eq) and HOBt (590 mg, 0.438 mmol, 0.47 eq) are successively added to a solution of 6-quinoline carboxylic acid (500 mg, 2.89 mmol, 3.1 eq), triethylamine (190 mg, 1.9 mmol, 2.0 eq), and 2,6-aminopyridine (102 mg, 0.93 mmol, 1.0 eq) in DMF (30 mL). The reaction mixture is then stirred at room temperature for 20 h. After removal of the solvent under reduced pressure, the residue is triturated in water (15 mL). The brown solid obtained by filtration was washed with Et_2O. After purification by column chromatography (silica gel, from DCM to 20% MeOH/DCM), a neutral bisquinoline derivative is obtained as a pale yellow solid (60 mg, 15.4% chemical yield). ^{1}H NMR (400 MHz, DMSO-d_6) δ 10.80 (s, 2H), 9.02 (td, J = 4.6, 1.7 Hz, 2H), 8.73 (d, J = 2.0 Hz, 2H), 8.54 – 8.48 (m, 2H), 8.34 – 8.24 (m, 2H), 8.12 (dd, J = 17.5, 8.9 Hz, 2H), 7.97 (s, 3H), 7.64 (ddd, J = 10.9, 8.2, 4.2 Hz, 2H).

EDL21[2]**:** A solution of the previously prepared bisquinoline derivative (60 mg, 0.143 mmol, 1.0 eq) in DMF (1 mL) and MeI (6 mL) is heated to 60°C under nitrogen and refluxed for 10 h. After cooling to room temperature, the formed precipitate is collected and dried by several Et_2O washings. After purification by preparative thin-layer chromatography (silica gel, MeOH/DCM/NH_3·H_2O=20:10:1), the amide-inversed bisquinolinium compound (**EDL21**) is obtained as an orange solid (10 mg, 9.94% chemical yield). ^{1}H NMR (400 MHz, DMSO-d_6) δ 11.20 (d, J = 9.2 Hz, 2H), 9.60 (d, J = 5.5 Hz, 2H), 9.38 (d, J = 8.4 Hz, 2H), 9.11 (dd, J = 4.2, 2.1 Hz, 2H), 8.80 – 8.71 (m, 2H), 8.68 – 8.61 (m, 2H), 8.36 – 8.24 (m, 2H), 8.06 – 7.97 (m, 2H), 7.95 (s, 1H), 4.68 (s,

6H). HR-MS(ESI) *m*/*z*: Calcd for [$C_{27}H_{23}N_5O_2I_2$-2I] 449.1841, found 449.1792.

1. Cheng, W.-C. *et al.* A combinatorial approach towards the synthesis of non-hydrolysable triazole–iduronic acid hybrid inhibitors of human α-L-iduronidase: discovery of enzyme stabilizers for the potential treatment of MPSI. *Chem. Commun.* 54, 2647–2650 (2018).
2. De Cian, A., DeLemos, E., Mergny, J.-L., Teulade-Fichou, M.-P. & Monchaud, D. Highly Efficient G-Quadruplex Recognition by Bisquinolinium Compounds. *J. Am. Chem. Soc.* 129, 1856–1857 (2007).

Table S1. Probes or dyes with the staining objective and different channel used in the assay and microscope filters and spectra settings. Related to STAR Methods.

Label	Excitation spectra (nm)	Emission spectra (nm)	Source	Cat-No	Stock concentration	Concentration used	Objective and Channel
Hoechst 33342	377/50	447/60	MCE	HY-15559	10 mg/ml	10 µg/ml	Nucleus
ROS Assay Kit-Highly sensitive DCFH-DA	488	500/50	DOJINDO	#R252	10 mmol/l	1:1000	ROS
TMRE	561	560-620	MCE	HY-D0985A	5 mmol/l	1:5000	MMP
mtSOX Deep Red-Mitochondrial Superoxide detection	633	640-700	DOJINDO	#MT14	10 mmol/l	1:1000	Mitochondrial ROS

Table S2. Primer Sequences used for qPCR. Related to STAR Methods.

Genes	Forward primer	Reverse primer
β-Actin	TCACCCACACTGTGCCCATCTACGA	CAGCGGAACCGCTCATTGCCAATGG
12S	GCTCGCCAGAACACTACGAG	CAGGGTTTGCTGAAGATGGC
COX1	AATAGGAGCTGTATTTGCCAT	AGAAAGTTAGATTTACGCCGAT
ND6	ATATACTACAGCGATGGCTA	AATCCTACCTCCATCGCTA

Methods S1: Detailed image analysis pipeline using Fiji and CellProfiler for single-cell quantification of ROS, mtROS, and MMP along with the synthesis protocol for compound EDL21, related to STAR Methods.

1 Materials and Methods

In this study, we developed a pipeline that combines high-content microscopy imaging, using different probes or stains (Table S1), with Fiji-based fluorescence quantification and CellProfiler-assisted cell painting to systematically investigate the effects of G4 ligands on mitochondrial health / mitochondrial membrane potential (MMP) with ROS and mtROS levels (Figure1).

1.1 Image Analysis Pipeline Using Fiji

Total ROS, mitochondrial-specific ROS (mtROS), and mitochondrial membrane potential (MMP), were simultaneously detected through high-content microscopy screening, followed by single-cell quantification using ImageJ/Fiji. Images containing nucleus staining and fluorescent marker signals were analyzed using an in-house macro-based single-cell analysis pipeline (Figure S14). We segmented cells via the nucleus staining channel and delineated cell profiles using the mtROS staining channel, saving these as regions of interest (ROIs). The fluorescence intensity across each channel for individual cells was then quantified, resulting in a data table capturing various single-cell parameters (e.g., area, mean intensity, etc.). Next, data mining and visualization was performed sequentially. A box plot illustrated the distribution of fluorescence intensities for G4 ligands at the single-cell level, with cisplatin serving as a positive control to induce ROS and reduce MMP. A 3D plot visualized the distribution of ROS, mtROS, and MMP across single cells in different treatment groups. Additionally, a heatmap demonstrated the correlations between ROS, mtROS, and MMP for each treatment group, comparing the impact of various compounds, including different G4 ligands and cisplatin, on mitochondrial homeostasis regulation with the normal context

(control) single-cell analysis Fiji-based Macro is attached in Data S1.

1.2 Image analysis pipeline-CellProfiler

1.2.1 Image quality control and preprocessing

We implemented the most stringent quality control during image acquisition. Images were captured using the HCA Live high-throughput live-cell imaging module (Brand: Nikon, Model: ECLIPSE Ji). Automated focusing was configured with custom settings, enabling real-time monitoring to ensure all images were captured on a clear focal plane. The calibrated high-content system ensured uniform illumination across the field of view, producing high-quality images. Acquisition parameters for each channel were optimized by setting positive and negative controls: under identical imaging parameters, the positive control showed moderate-to-high expression without signal saturation, while the negative control exhibited no distinct fluorescent signal. This protocol guaranteed the authenticity and reliability of subsequent quantitative analysis. Only curated, high-quality images were imported into CellProfiler. Given the success of the upfront acquisition QC, we intentionally adopted a minimalist CellProfiler pipeline focused on feature extraction. We relied on the high baseline quality of the input images and the robustness of CellProfiler's default segmentation algorithms, such as IdentifyPrimaryObjects. Subsequently, the structure extraction pipeline for CellProfiler was configured based on the phenomics approach*[19]* described in the literature that utilized CellProfiler.

Then images were processed and analyzed with the open-source image analysis software CellProfiler (available at https://cellprofiler.org/), version 4.2.4.

1.2.2 Segmentation

Segmentation (Figure S15) based on the Hoechst channel was performed by applying a Gaussian blur followed by Otsu thresholding to segment the outlines of each nucleus. The cell objects were segmented using Watershed segmentation using Otsu thresholding, using the nuclei as a seed. The cytoplasm cell compartment was defined as the cell object subtracted from the nuclei.

1.2.3 Image analysis

In the pipeline, the nuclei were identified using the “IdentifyPrimaryObjects” module then expanded to represent the cell bodies. Fluorescence staining was also identified with the “IdentifySecondaryObjects” module. Raw channel images were rescaled for accurate fluorescence staining and background intensity measurements that were obtained using the “MeasureObjectIntensity” and “MeasureImageIntensity” modules, respectively.

1.2.4 Feature extraction

After identification of the four cell compartments, phenotypic characteristics were measured using the AreaShape, Correlation, Intensity, Granularity, Location, Neighbors and RadialDistribution modules as provided by CellProfiler. A total of 541 features were extracted from each object which were exported into csv format for downstream analysis. For each condition, more than 400 cells were analyzed. All feature definitions can be found in the supplementary files Data S2, containing all extracted cellular features in HeLa cells, and Data S3, containing all extracted cellular features in HPFC cells. respectively. Key categories of parameters include:

1. Morphological features
    - AreaShape_Area: Cell area, directly reflecting cell size.
    - AreaShape_Perimeter: Cell perimeter, associated with shape complexity.
    - AreaShape_Eccentricity: Eccentricity, indicating the degree of elongation.
    - AreaShape_FormFactor: Shape factor, with values closer to 1 indicating a more circular morphology.
    - AreaShape_Solidity: Solidity, reflecting the convexity of the cell outline.
2. Intensity features
    - Intensity_MeanIntensity_*: Mean fluorescence intensity in each channel, indicating the average expression level of the marker.
    - Intensity_IntegratedIntensity_*: Integrated intensity, calculated as mean intensity × area, representing total marker abundance.
3. Texture features
    - Texture_Contrast_*: Contrast, measuring local intensity variation.
    - Texture_Entropy_*: Entropy, reflecting image complexity.
    - Texture_Correlation_*: Correlation, representing linear dependence of pixel gray levels.

4. Granularity features
    - Granularity_*: Granularity measures indicating the characteristic scale of intracellular structures.
5. Neighborhood features
    - Neighbors_NumberOfNeighbors: Number of neighboring cells, reflecting local cell density.
    - Neighbors_FirstClosestDistance: Distance to the nearest neighbor, indicating spatial relationships between cells.
6. Correlation features
    - Correlation_Correlation_*: Cross-channel intensity correlations, potentially reflecting co-localization patterns.

1.2.5 Feature analysis

To compare morphological profiles, the features were presented as cluster Heatmaps. Feature normalization ensured that each single cell feature (clustermap column) had a mean of 0 and variance of 1. We performed Heatmap and PCA analysis based on all single-cell features from the analyzed images of each group after removing the first three columns, as shown in the supplementary files named Data S2 and Data S*3*. The normalized features were aggregated to a mean profile per compound. Unsupervised hierarchical clustering on the morphological profiles was computed using the Euclidean metric, and the clustering algorithm using Ward's method and visualized using ComplexHeatmap R package using R (version 4.3.1). Pearson's correlation coefficients were pairwise calculated using Panda's python library function with default settings. Visualization of the high dimensional data was done using the principal component analysis (PCA) on the centred and normalized features and factoextra R package using R studio (version 4.3.1). As shown in Figure S7, our analysis focused on the first two principal components (PC1 and PC2). We subsequently examined the variables with the highest loadings on each component, revealing that the major contributors were features related to Texture and Intensity.

The R Studio scripts used for heatmap generation and principal component analysis are provided in the Supplementary Files : Heatmap R studio macro(Data S4) and PCA5 R studio macro(Data S5).

1.3 Synthesis and Characterization of EDL21

All chemicals and solvents used for synthesis were purchased from commercial suppliers (Adamas, Jingshan Chemical Reagent, TCI). Thin-layer chromatography (TLC) was used to monitor all reactions. Flash chromatography was carried out using silica gel (200–300 mesh). ^{1}H NMR spectra were recorded using a Varian 400 MHz NMR at 25°C, with dimethyl sulfoxide (DMSO) as the internal standard. High-resolution mass spectra (HR-MS) was performed on a Bruker BioTOF IIIQ system. They were synthesized and characterized by the following methods.

The structure of EDL21 was prepared with the synthetic routes as shown in Scheme S1. They were characterized by ^{1}H NMR spectra and High-resolution mass spectra. The raw NMR and Highresolution mass spectra dataset for EDL21 has been deposited in the Biological Magnetic Resonance Data Bank (BMRB) under accession number bmrbig144.

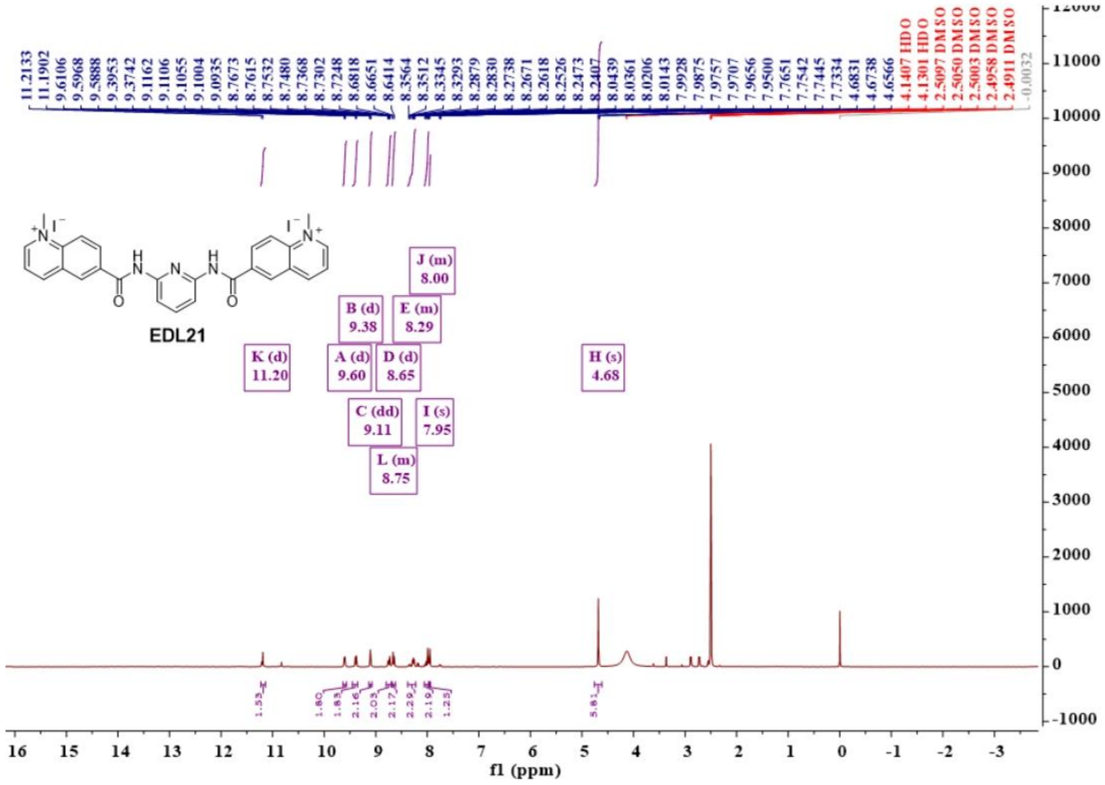

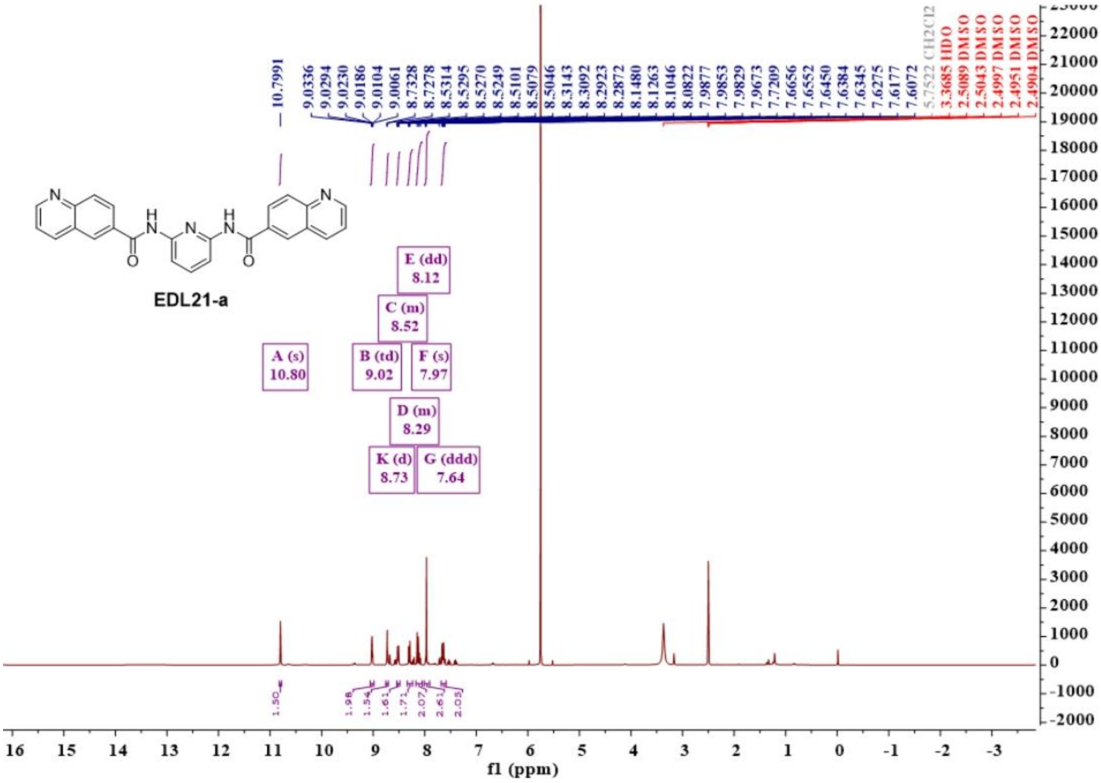

EDL21-a
A (s) 10.80
B (td) 9.02
C (m) 8.52
D (m) 8.29
E (dd) 8.12
F (s) 7.97
G (ddd) 7.64
K (d) 8.73
10.7991
9.0336 9.0294 9.0230 9.0186 9.0104 9.0061 8.7328 8.7278 8.5314 8.5295 8.5270 8.5249 8.5101 8.5079 8.5046 8.3143 8.3092 8.2923 8.2872 8.1480 8.1263 8.1046 8.0822 7.9877 7.9853 7.9829 7.9673 7.7209 7.6656 7.6552 7.6450 7.6384 7.6345 7.6275 7.6177 7.6072
5.7522 CH2Cl2
3.3685 HDO
2.5089 DMSO 2.5043 DMSO 2.4997 DMSO 2.4951 DMSO 2.4904 DMSO
f1 (ppm)

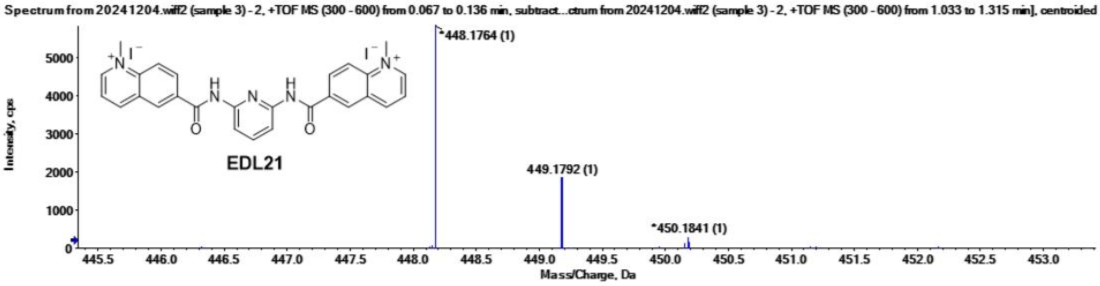

Spectrum from 20241204.wiff2 (sample 3) - 2, +TOF MS (300 - 600) from 0.067 to 0.136 min, subtract...ctrum from 20241204.wiff2 (sample 3) - 2, +TOF MS (300 - 600) from 1.033 to 1.315 min], centroided
EDL21
448.1764 (1)
449.1792 (1)
450.1841 (1)
Intensity, cps
Mass/Charge, Da
