# Supplementary figures and images for: Do G4 ligands induce mitochondrial dysfunction without ROS induction
Source: iScience. 2026 May 22;29(6):116036. doi: 10.1016/j.isci.2026.116036 (PMC13224012; doi:10.1016/j.isci.2026.116036)

Full unedited western blots

Figure 4D

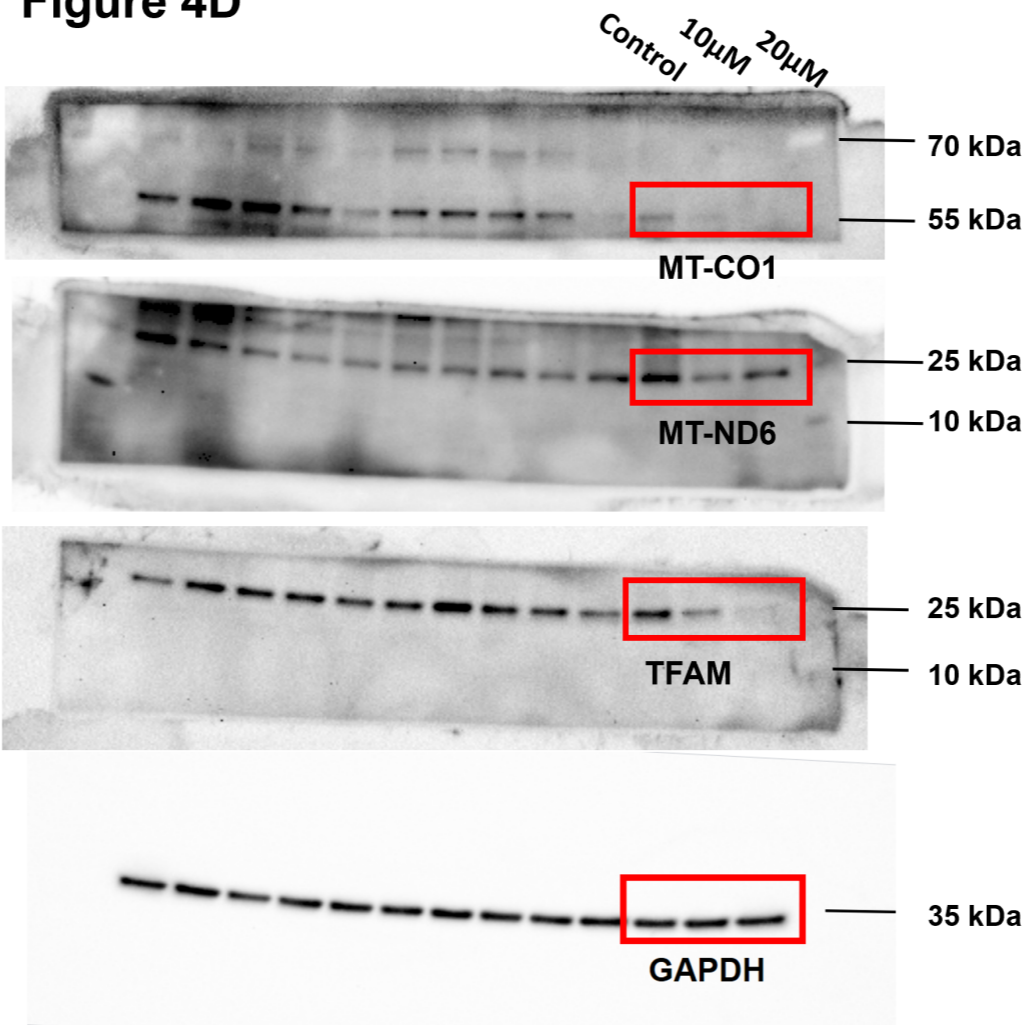

Supplement: Data S6. Full unedited wastern blots [file mmc8.pdf]
